# Machine Learning Approach for Improved Longitudinal Prediction of Progression from Mild Cognitive Impairment to Alzheimer’s Disease

**DOI:** 10.3390/diagnostics14010013

**Published:** 2023-12-20

**Authors:** Robert P. Adelson, Anurag Garikipati, Jenish Maharjan, Madalina Ciobanu, Gina Barnes, Navan Preet Singh, Frank A. Dinenno, Qingqing Mao, Ritankar Das

**Affiliations:** Montera, Inc. dba Forta, 548 Market St, PMB 89605, San Francisco, CA 94104-5401, USA; robert.adelson@fortahealth.com (R.P.A.); agarikipati@fortahealth.com (A.G.); jmaharjan@fortahealth.com (J.M.); mciobanu@fortahealth.com (M.C.); gbarnes@fortahealth.com (G.B.); nsingh@fortahealth.com (N.P.S.); fdinenno@fortahealth.com (F.A.D.); ritankar@fortahealth.com (R.D.)

**Keywords:** mild cognitive impairment, Alzheimer’s disease, machine learning, disease progression

## Abstract

Mild cognitive impairment (MCI) is cognitive decline that can indicate future risk of Alzheimer’s disease (AD). We developed and validated a machine learning algorithm (MLA), based on a gradient-boosted tree ensemble method, to analyze phenotypic data for individuals 55–88 years old (*n* = 493) diagnosed with MCI. Data were analyzed within multiple prediction windows and averaged to predict progression to AD within 24–48 months. The MLA outperformed the mini-mental state examination (MMSE) and three comparison models at all prediction windows on most metrics. Exceptions include sensitivity at 18 months (MLA and MMSE each achieved 0.600); and sensitivity at 30 and 42 months (MMSE marginally better). For all prediction windows, the MLA achieved AUROC ≥ 0.857 and NPV ≥ 0.800. With averaged data for the 24–48-month lookahead timeframe, the MLA outperformed MMSE on all metrics. This study demonstrates that machine learning may provide a more accurate risk assessment than the standard of care. This may facilitate care coordination, decrease healthcare expenditures, and maintain quality of life for patients at risk of progressing from MCI to AD.

## 1. Introduction

Mild cognitive impairment (MCI) is cognitive decline that is atypical for an individual’s age [1,2]. Up to 18% of individuals over 60 years of age experience MCI [3]. MCI may be the result of Alzheimer’s disease (AD), a type of dementia, or alternative etiologies, including conditions for which cognitive decline can be reversed with appropriate treatment. AD is an irreversible neurodegenerative disease that causes deterioration and impairment in cognition, memory, and functioning, and is ranked among the top five causes of mortality among all United States (US) adults aged 65 years and older [4].

The risk of progression from MCI to dementia (including AD) increases over time, with approximately 10% of individuals progressing within one year following MCI diagnosis, and 80% progressing within six years following MCI diagnosis [5]. Individuals who progress from MCI to AD face severe cognitive deterioration that ultimately eliminates the ability to independently engage in activities of daily living without reliance on caregivers [6]. Individuals with AD require increasingly intensive and costly care, estimated at approximately USD 56,000 per individual per year [7].

The ability to stratify patients with MCI who are likely to progress to AD is essential for optimizing clinical and non-clinical care coordination in advance of severe disease onset, which may allow for improved quality of care, quality of life, decreased healthcare expenditures, and a reduction in health and safety risks associated with AD [4]. For patients that are not at risk for progressing from MCI to AD, a differential diagnosis can prompt treatment to delay or reverse cognitive decline [3].

Assessing a patient’s risk of progressing from MCI to AD is an inherently complex process that involves a full clinical assessment and collecting patient performance data from assessment scales that measure functioning in specific domains [8,9]. Standard of care (SOC) assessment scales for measuring cognitive function in patients with MCI as a method of predicting risk of progression to dementia include the Montreal Cognitive Assessment (MoCA) and the mini-mental state examination (MMSE), the latter of which is widely used in clinical practice to monitor progression of MCI to AD and is considered the gold-standard for measuring cognitive impairment [10]. Other methods for assessing risk include genetic testing for individuals aged 60 and younger, neuroimaging, and biomarker analysis [11,12]. However, these methods are limited by their invasive nature, prohibitive cost, and the need to obtain serial measurements over time to ensure accuracy and observe longitudinal cognitive changes [13].

Artificial intelligence (AI) and machine learning (ML) decision support tools have been validated for a variety of clinical predictive tasks, including those related to neurodegenerative and neurodevelopmental conditions as well as other acute and chronic conditions [14,15,16,17,18,19,20,21,22,23,24,25,26,27,28,29]. These tools provide the opportunity for earlier detection of medical conditions or complications and may, in turn, facilitate earlier treatment when appropriate for a particular condition. Many of these tools used state-of-the-art methods for their classification tasks, including a range of input features varying from complex biomarkers to assessment data and diverse ML models (Supplementary Table S1). Hinrichs et al. used ML to analyze a combination of imaging data, biological data, and cognitive assessment testing data, in order to classify AD patients versus individuals with typical cognitive ability (accuracy of 0.924) and to predict which patients with MCI would progress to AD versus reverting to typical cognition (area under the receiver operating characteristic curve/AUROC of 0.9708) [17]. A multi-kernel learning algorithm was used as the basis for their ML model, which facilitated analysis of complex and diverse types of data while limiting the complexity of the model itself. Massetti et al. employed a random forest ML model to predict progression from MCI to AD using neuropsychological evaluation results and AD biomarkers and achieved an accuracy of 0.86 [20]. Noting that the presence of emergent neuropsychological disorders correlates with progression from MCI to dementia, Mallo et al. used nine ML models to evaluate symptoms of these disorders in patients with MCI to establish risk of progression to dementia and achieved an accuracy of 0.88 by using a random forest model [27]. Morabito et al. examined resting state electroencephalographic (EEG) waveform data to measure degeneration of connectivity density within the brains of individuals with AD and yielded statistically significant (*p* < 0.05) changes between initial assessment and follow-up at 3 months, indicating that AD progression can be measured by these changes in EEG data [25]. Rutkowski et al. employed wearable EEG headsets constituting a brain–computer interface to analyze participants’ responses to memory and visuospatial cognitive tasks [28]. Supervised and unsupervised ML methods were used to analyze the data and differentiate between patients with typical cognition and patients with MCI, yielding AUROCs ranging from 0.49 to 1.00 depending on the region of the brain that was analyzed, but the latter results are not likely to be generalizable. Spasov et al. employed a deep learning approach and novel methods in which the ML model concurrently learned two classification tasks: classifying participants with MCI who would progress to AD and classifying participants as having AD or typical cognition (i.e., control group) [29]. Data were derived from imaging, demographics, neuropsychological assessments, and genetic data and the model achieved an AUROC of 0.925 for predicting MCI conversion to AD within 3 years and AUROC of 1.00 for differentiation between participants with AD and those with typical cognition. Marcisz and Polanska used a logistic regression model to classify individuals as having MCI, AD, or normal cognition using magnetic resonance imaging radiomic data to evaluate brain volume and yielded the highest AUROC (0.997) for classification of AD and the lowest AUROC (0.793) for classification of MCI vs. normal cognition [30]. Peng et al. predicted the progression of MCI to AD using feature inputs to analyze white matter from the brain measured by positron-emission computed tomography (PET) scans [31]. Multimodal data were used as input features to construct an ML model, which yielded an AUROC of 0.865 within the test set for prediction of individuals with MCI converting to AD. Chen et al. used Orthogonal Latent space learning with Feature weighting and Graph learning (OLFG) to evaluate atrophy in various regions of the brain and achieved AUROCs of 0.719, 0.970, and 0.814 for distinguishing between normal cognition vs. MCI, normal cognition vs. AD, and non-progressive MCI vs. progressive MCI, respectively [32]. Martínez-Torteya et al. used logistic regression to analyze multimodal data inputs, including imaging, clinical measurements, and results from biological testing to distinguish between individuals with AD, MCI, and normal cognition and yielded AUROCs of 0.945, 0.864, and 0.838 for distinguishing between normal cognition and AD, normal cognition and MCI, and MCI and AD, respectively [33]. Despite the relatively good performance of these ML models for their respective classification tasks, each has critical limitations. For example, invasive inputs that are expensive to acquire were required to make predictions (biomarkers, imaging, genetic data, etc.) and models were trained on biased data, thus limiting generalizability. Additionally, with the exception of the study by Spasov et al., these studies do not provide for longitudinal prediction of progression of MCI to AD in future time windows [29].

Given the lack of efficient and reliable methods to determine a patient’s risk of progressing from MCI to AD, there is a need to develop and validate advanced longitudinal prediction methods for MCI progression to AD. With the ability to analyze the multitudinous amount of individualized data that is accrued over time in electronic health records (EHRs), ML can uncover latent patterns in data to enable personalized and accurate risk assessments.

**Figure 1:**
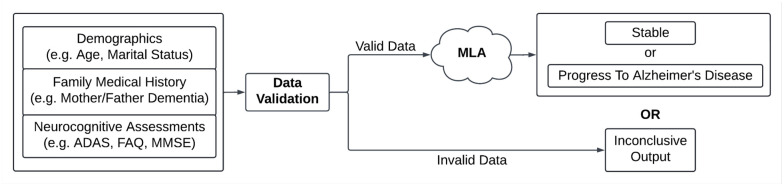
Diagram of inputs and outputs for the machine learning algorithm (MLA). Abbreviations: Alzheimer’s Disease Assessment Scale (ADAS), Functional Activities Questionnaire (FAQ), mini-mental state examination (MMSE).

Here, we describe the development and validation of a machine learning algorithm (MLA) to predict MCI to AD progression. A widely used gradient-boosted tree ensemble method, XGBoost (version 1.6.2), was used to develop our MLA [34]. We assessed the MLA’s performance in each of the 12-, 18-, 24-, 30-, 36-, 42-, and 48-month prediction windows, and then used averaged data (24–48-month lookahead timeframe) to examine the performance of the MLA. The performance of our MLA was compared with the performance of the MMSE SOC and of three additional popular machine learning models which we trained on substantially the same dataset used for training the MLA, *k*-nearest neighbors (KNN), a multi-layer perceptron (MLP) neural network, and logistic regression (LR). The results of this exploratory study indicate that an ML-based risk assessment tool can serve as a personalized risk assessment approach with excellent performance. Figure 1 depicts the workflow of the MLA in a clinical setting. Used for clinical decision support, this MLA may provide the opportunity for healthcare providers (HCPs) to conduct broad screenings using fewer resources and with better performance than other risk assessment methods.

## 2. Materials and Methods

Retrospective data from the Alzheimer’s Disease Neuroimaging Initiative (ADNI) (adni.loni.usc.edu) was used for our analysis [35]. ADNI was launched in 2003 and its primary goal has been to test whether serial MRI, positron emission tomography (PET), other biological markers, and clinical and neuropsychological assessments can be combined to measure the progression of MCI and early AD. ADNI consists of phenotypic data on more than 2000 individuals, aged 55 years and older. As data were de-identified to maintain compliance with Health Insurance Portability and Accountability Act (HIPAA), this research did not constitute human subjects research per the 45 US Code of Federal Regulations 46.102.

**Figure 2:**
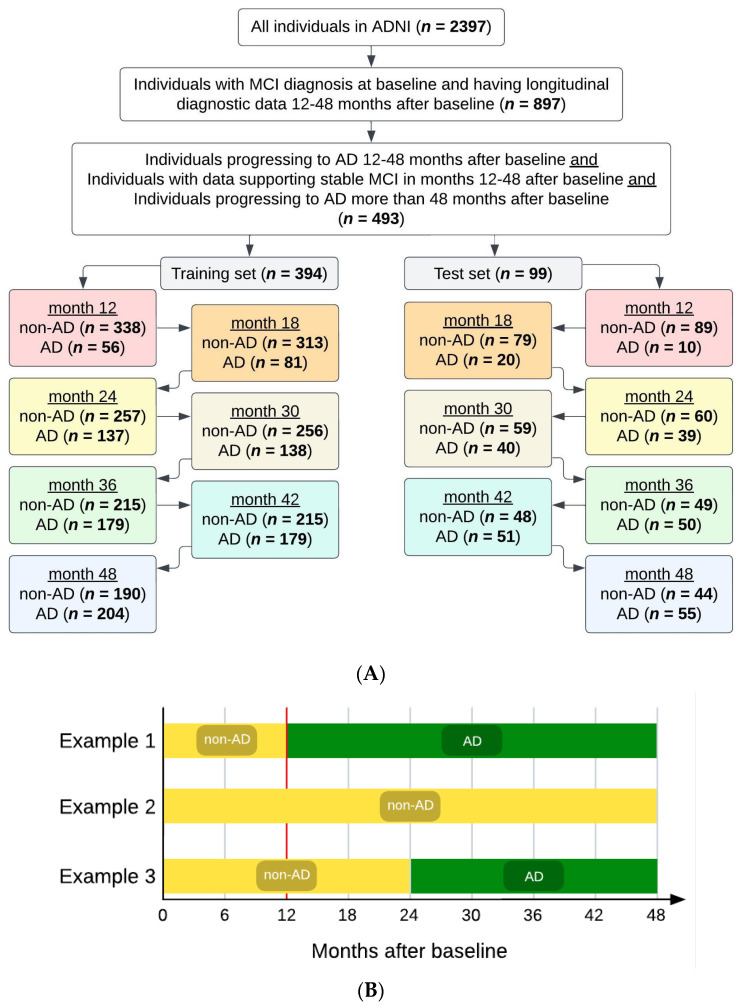
(**A**) Attrition chart for the dataset used for modeling. We selected individuals receiving a mild cognitive impairment (MCI) diagnosis at their baseline visit in the Alzheimer’s Disease Neuroimaging Initiative (ADNI) study. Selection criteria included data availability to confirm MCI stability or progression to Alzheimer’s disease (AD). The filtered dataset was split 80/20 into training and test sets, respectively. (**B**) Examples of subjects’ diagnostic statuses/tags over the course of the study. The vertical red line is at month 12, the first prediction timepoint/window investigated, before which all subjects were filtered to have MCI (not AD).

Data were filtered to ensure individuals in the dataset had the necessary inputs, as shown in Figure 2A. From the 2397 total individuals in the dataset, we selected the individuals (*n* = 897) who had (i) an MCI diagnosis at baseline (the beginning of the ADNI study), with no AD diagnosis at that time; and (ii) longitudinal diagnostic data for at least 12–48 months from baseline. Then, we selected the individuals with desired phenotypic data (*n* = 493) as follows: 259 individuals who received an AD diagnosis at some point between 12 and 48 months from baseline were filtered into the AD cohort, and 234 individuals who did not receive an AD diagnosis at or before 48 months from baseline were filtered into the non-AD cohort. The non-AD cohort included individuals with data supporting stable MCI in months 12–48 after baseline, as well as individuals progressing to AD more than 48 months after baseline (i.e., the progression from MCI to AD occurred beyond 48 months from baseline). Further, the non-AD cohort excluded individuals with insufficient diagnostic data to indicate stable MCI (i.e., no confirmation of MCI status at month 48 and no diagnostic information after month 48). Diagnosis of AD or MCI within the database was reported by HCPs and was considered the ground truth to identify the positive (AD) and negative (non-AD) classes of patients. For diagnosing AD within ADNI, key factors were presence of memory complaints, MMSE score (under 27 points), Clinical Dementia Rating (CDR) score (0.5 or 1), and Logical Memory II subscale of the Wechsler Memory Scale–Revised (score up to 25, depending on education level) [36].

Comorbidity information regarding diabetes, cerebrovascular disease, coronary heart disease, depression, hypertension, bone and musculoskeletal diseases (osteoporosis and osteoarthritis), and hearing and vision impairment were extracted from medical history files which were collected as part of the ADNI study. These comorbidities have been associated with AD as risk factors, frequently co-occur with AD, or are a result of AD [37,38,39].

### 2.1. Multiple Prediction Windows/Timepoints

After filtering, the study cohort (*n* = 493) was randomly split into a training set and a hold-out test set (i.e., test set), such that 80% of the individuals (*n* = 394) were in the training set and the remaining 20% of the individuals (*n* = 99) were in the test set. The training and test sets remained completely independent of each other (i.e., no overlap between the individuals in the training set and those in the test set). Maintaining separation between the training and test sets prevented data leakage from the training set from affecting the MLA’s performance evaluation.

Each individual in the filtered study cohort (*n* = 493) was given a tag at each of the 7 timepoints under consideration (months 12, 18, 24, 30, 36, 42, and 48), with the given tag corresponding to the individual’s status (AD or non-AD) at that particular timepoint in the study. The same 394 individuals constituted the training set at each of the 7 studied timepoints. Similarly, the same 99 individuals constituted the test set at each of the 7 studied timepoints. While the individuals in each of the training and test sets did not change between the seven studied timepoints, their tags could change in some cases, as follows. Once categorized as AD for a given timepoint, an individual retained the AD tag for all future timepoints. However, the tag of a non-AD individual could change into an AD tag at a subsequent timepoint, should the patient have developed AD since the previous timepoint. For example, and as shown in Figure 2B, an individual could have a non-AD tag at month 12, continue to have a non-AD tag at month 18, but then the same individual could acquire the AD tag at month 24, and this AD tag would remain with this particular individual at 30, 36, 42, and 48 months. As another example, an individual could receive a non-AD tag at month 12 and continue to have this non-AD tag at 18, 24, 30, 36, 42, and 48 months. As yet another example, an individual could receive the AD tag at month 12, and continue to have this AD tag at 18, 24, 30, 36, 42, and 48 months.

The demographics for each of the training sets were substantially consistent between different timepoints, and representative training set demographic information is shown in Table 1 for the 12-, 24-, and 48-month timepoints. The demographic information for the other timepoints (18, 30, 36, and 42 months) is given in Supplementary Table S2. The age in the demographics tables is given for the baseline of the study, i.e., 12 months prior to the first time point (the 12-month timepoint or prediction window). The demographics for each of the test sets were also substantially consistent between different timepoints, and test set demographic information is given in Supplementary Tables S3 and S4 for all seven timepoints (12, 18, 24, 30, 36, 42, and 48 months).

The MLA was trained at each of the seven timepoints, with the training set having the number of AD and non-AD individuals as indicated in Figure 2A, and then the trained MLA was analyzed at the time window where it was trained on the corresponding test set to evaluate model results to determine the algorithm’s efficacy at that particular timepoint. For example, at the 36-month timepoint, the MLA was trained on data from 394 individuals (215 non-AD, 179 AD), and then was tested on the corresponding test set: 99 individuals (49 non-AD, 50 AD).

As the MLA was distinctly trained at each of the seven timepoints, a type of forward feature selection algorithm was applied separately at each of the 7 timepoints (12, 18, 24, 30, 36, 42, and 48 months). For any given timepoint, feature selection involved reading in and combining the screening and baseline data for each individual, using their status (AD or non-AD) at that timepoint as their label for feature selection, removing one out of each pair of highly correlated features (Pearson correlation coefficient > 0.85), and checking feature importance for 5 to 50 features per iteration of feature selection. For a given iteration of feature selection, the single feature AUROC was first calculated for each feature, followed by calculating a pairwise AUROC for each combination of features. The features with the highest single feature AUROC, and the features with the highest pairwise AUROC were placed in the list of most important features. This process continued until at most 50 features were chosen for that particular iteration of feature selection, with fewer features chosen if fewer than 50 features contributed meaningfully to the prediction, a threshold determined through parameter tuning prior to selecting features. The features that were used in forward feature selection at each of the seven timepoints are shown in Table 2, with detailed breakdown about the components for each input category shown in Supplementary Table S5.

Some feature processing was performed prior to inputting the features into the prediction algorithm. For example, categorical features such as lists of comorbidities were encoded to binary features indicating the presence or absence of a specific comorbidity. The remaining features were utilized “as-is” without any processing methods to alter the raw data value based on the use of a gradient-boosted tree-based model, which does not require data normalization or other preprocessing. Tree-based models split data for each feature individually, eliminating the need for data normalization or standardization. Input features were not explicitly combined or compared outside of the prediction algorithm. However, the input features were implicitly compared to thresholds and combined by the prediction algorithm to provide the final output. These thresholds were learned by the prediction algorithm during the training process.

As discussed, the training process was performed on each training set for a particular timepoint. During the training process, the gradient-boosted tree algorithm XGBoost was utilized for the classification of individuals having AD vs. non-AD individuals. This XGBoost-based model combines the estimates of simpler, weaker models—in this case, shallow decision trees—to make predictions for a chosen target [34]. Tree models utilize the values of a subset of the inputs to build a path to the class (e.g., AD class, non-AD class) to which a particular set of inputs belongs, with the path being known as a decision tree. This was repeated to develop a series of decision trees which were utilized together to determine the final output of the MLA. Due to the MLA containing several trees, each with a subset of the input data, the MLA was able to perform classifications that accounted for the heterogeneity of feature values with which an individual diagnosed with AD may present. One of the benefits of using XGBoost is that it has been shown to perform better than other ML models on tabular data, which made it an appropriate choice for our dataset [40]. We performed a grid search in order to select the best combination of hyperparameters of the XGBoost model utilized in the MLA. The grid search method evaluates a range of hyperparameter values in order to identify the combination of hyperparameters which optimizes model performance. This tuning process led to selection of the following hyperparameter values: a learning rate of 0.1, a maximum tree depth of 2, inclusion of 200 estimators in the model, and use of a logarithmic loss evaluation metric for validation data [40].

### 2.2. Data Averaging for a 24–48-Month Lookahead Timeframe

We also investigated whether averaging across different prediction windows would improve the prediction uncertainty that results from noise in the training data, where the noise is owed to an imperfect deterministic relationship between input features and diagnostic ground truth. This type of training data noise can also impact feature selection for a given prediction window, and we sought to minimize the effect of training data noise by averaging prediction outputs across different prediction windows. The goal was to determine the effect of such averaging on the performance metrics. For this, the unrounded predictions from the 24-, 30-, 36-, 42-, and 48-month windows were averaged for the same test set of 99 individuals from Figure 2 to yield an averaged MLA prediction. The predictions at the 12- and 18-month windows were excluded when averaging the data, because the AUROC values were not significantly different between the MLA and MMSE for these timepoints, whereas the AUROC was significantly higher for the MLA than for the MMSE model starting at the 24-month prediction window. The MMSE was not designed to be used for long-term longitudinal predictions; thus, it was expected that it would perform relatively well at the shorter-term timepoints (12 and 18 months) [41]. However, a reliable prediction tool for the longer-term timepoints is lacking.

### 2.3. SOC Comparator

We compared the metrics produced by our MLA with the metrics of an SOC screening tool, the MMSE. The MMSE is widely used in clinical practice as a screening device for cognitive impairment, with a low score suggesting the need for further evaluation. Its ubiquitous, routine use by medical practitioners makes the MMSE a valuable comparator for more complex algorithmic approaches such as the MLA we developed. Given its clinical importance, components of the MMSE were included as input features in our MLA. This was similar to the approach of Marcisz and Polanska, who trained a linear regression model on a small set of features including the MMSE to investigate AD but did not factor in changes in disease status over time and did not include other neuropsychological tests and important comorbidities in building their model. They similarly used the MMSE on its own as a base comparison model [30]. In our MLA, MMSE input features allowed for the comparison of the predictive performance of the MMSE alone to a model built using MMSE components along with other features of interest, rather than comparing the MMSE to a model built solely using other features. This is important because, from a practical perspective, the MLA does what the MMSE could never do on its own: it delivers powerful predictions longitudinally up to 48 months. The MLA practically accounts for the SOC, rather than excluding the SOC solely to allow for a theoretical comparison to the MMSE.

It should be noted that the MMSE classifier is not based on ML, and this classifier has been generated by splitting the dataset corresponding to each timepoint into AD-positive and AD-negative classes based on MMSE score thresholds, as follows. The MMSE total score has possible values ranging from 0 to 30. MMSE total score values between 0 and 30 were used as a threshold (i.e., the MMSE score value below which an individual can be classified as having AD) for separating the positive and negative classes, resulting in a set of sensitivity and specificity values corresponding to that particular threshold, as illustrated by the resulting receiver operating characteristic (ROC) curves for the MMSE classifier in Supplementary Figure S1. All individuals in the filtered study cohort (*n* = 493) have MMSE scores between 19 and 30; thus, there are only 12 possible MMSE score values in the dataset. The individuals in each test set displayed only 7 of these 12 possible values; thus, each MMSE classifier ROC curve has 7 data points. A different MMSE classifier ROC curve was generated for each of the seven timepoints (12, 18, 24, 30, 36, 42, and 48 months). It should be noted that while the individuals in the test set remained the same (*n* = 99) between the different timepoints, the data for each individual may have changed from one timepoint to another. Specifically, at each of the seven timepoints (months 12, 18, 24, 30, 36, 42, and 48), each individual in the filtered study cohort (*n* = 493) was given a tag corresponding to the individual’s status (AD or non-AD) at that particular timepoint in the study.

### 2.4. Comparison with Other Machine Learning Models

To gauge the performance of the XGBoost-based MLA versus models built with other machine learning algorithms, we trained three additional models for comparison. We selected one model which is substantially simpler compared to XGBoost, *k*-nearest neighbors (KNN), one which is substantially more complex, a multi-layer perceptron (MLP) neural network, and one based on a logistic regression approach previously published in the context of the ADNI dataset [30].

KNN is a non-parametric, supervised learning classifier which uses a proximity metric to classify individual samples into two or more classes [42]. This is a simple, instance-based approach which performs best on low-dimensional data. The classifier algorithm evaluates the vector distance between an input and each of the training examples to determine which examples are closest in distance to the input. Amongst classifications of the *k* nearest neighbors, a predicted classification is selected for the input. Following hyperparameter tuning, we set *k* = 7 as the number of nearest neighbors to use in classification.

An MLP is a feedforward artificial neural network model which maps the input dataset to a set of outputs, in this case in a binary manner as this is a binary classification [43]. The network consists of a series of dense hidden layers which extract information from the data followed by a final softmax layer which scales outputs into a binary prediction. Following hyperparameter tuning, we used three hidden layers (in order from first to last, containing 150, 100, and 50 neurons), the ‘Adam’ algorithm for weight optimization, a maximum of 300 epochs (the number of times each data point can be used), and the rectified linear unit function as the activation function for the hidden layer.

LR is a generalized linear modeling approach for binomial and multinomial classification tasks, commonly used in medicine and epidemiology [44]. In this case, a binary outcome (stable at MCI or progression to AD) was assumed, on the condition of the input features, to follow a binomial distribution. This approach assumes a linear relationship between the logarithm of the odds of the predicted outcome and the predictors (input features). In this case, we used LR for binary classification of a dataset with a relatively small number of features, so hyperparameter tuning was not performed.

The same set of features output from feature selection for the MLA at each time window were also utilized for the three comparison models in order to allow for a more direct comparison rather than introducing another complexity to the comparison. The KNN model, MLP model, and LR model required additional data processing, compared to the data processing carried out for the XGBoost-based MLA, to successfully train these additional comparison models. In contrast to XGBoost models, KNN, MLP, and LR cannot handle null values implicitly; thus, any data points with null values either need to be removed from the dataset or replaced (e.g., using imputation). In these cases, individuals with missing data were simply removed from the dataset before proceeding.

### 2.5. Statistical Analysis

The predictive performance of each trained model (for each of the 7 prediction windows, as well as for the averaging approach) on its corresponding test set was evaluated using several standard performance measures [45,46]. These performance metrics were AUROC (the probability that a classifier will be able to distinguish between an instance of the positive class and one of the negative class), sensitivity (true positive rate), specificity (true negative rate), positive predictive value (PPV, the proportion of true positives among all positive predictions), negative predictive value (NPV, the proportion of true negatives among all negative predictions), and accuracy (the proportion of correct predictions among all predictions).

The 95% confidence intervals (CIs) for AUROC were calculated using a bootstrapping method. For the bootstrapping method, a subset of patients from the hold-out test dataset were randomly sampled and the AUROC was calculated using the data from those patients. This step was repeated 1000 times with replacement. From these 1000 bootstrapped AUROC values, the middle 95% range was selected to be the 95% CI for the AUROC. As the sample size of the hold-out test dataset was greater than 30, the CIs for the other performance metrics were calculated using normal approximation.

### 2.6. System Requirements

In this study, we utilized a 2021 MacBook Pro with 16 GB of memory and an Apple M1 Pro chip, for training, testing, and performance evaluation. Training was completed in less than three seconds for each model at each time window. Since compute capacity is not a limiting factor for machine learning analysis of tabular data, most modern Mac, Windows, and Linux computers with at least 8 GB of memory can easily handle training any of the models (XGBoost-based, KNN, MLP, and LR) we describe here.

All data processing, training, testing, and evaluation were performed in Python. Only commonly used packages were utilized: pandas for data input and manipulation, scikit-learn for machine learning using the three comparison models (KNN, MLP, and LR), XGBoost for machine learning using the MLA, NumPy for working with arrays, and Matplotlib for creating visualizations [47,48,49,50,51].

## 3. Results

**Figure 3:**
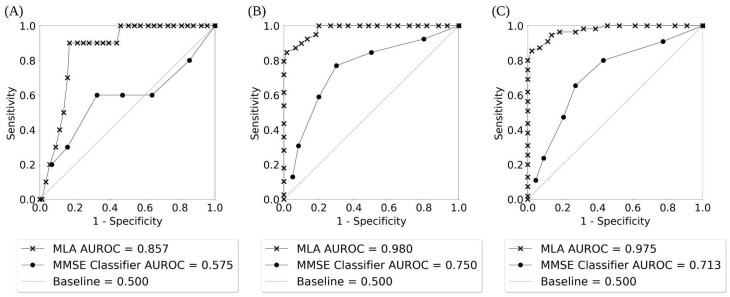
The receiver operating characteristic (ROC) curves for AD class vs. non-AD class as given by the MLA and the MMSE classifier showing the performance of the MLA and the MMSE over time: (**A**) 12-month prediction window; (**B**) 24-month prediction window; (**C**) 48-month prediction window.

Figure 3 displays the ROC curves for identification of progression vs. non-progression from MCI to AD for 12-, 24-, and 48-month prediction windows (with months 18, 30, 36, and 42 shown in Supplementary Figure S2). The ROC curves show the true positive rate or sensitivity against the false positive rate or 1-specificity, and illustrate the diagnostic ability of a binary classifier at different thresholds. We additionally included a baseline curve (dashed line) for a classifier providing no predictive value, for the purpose of comparison. The legend in Figure 3 shows the individual AUROC values for the MLA and the MMSE classifier, as well as the baseline. Supplementary Figure S3 shows the ROC curves for AD class vs. non-AD class as given by the MLA classifier when the prediction algorithm is tested on the hold-out test sets for each of the seven prediction windows showing the performance of the MLA over time.

The performance of the MLA, along with the performance of three comparison models (KNN, MLP, and LR) and the SOC, for differentiating between the AD class and the non-AD class is shown in Table 3 for the 12-, 24-, and 48-month prediction windows (with months 18, 30, 36, and 42 shown in Supplementary Table S6). The MLA performance metrics in Table 3 and Supplementary Table S6 were obtained by selecting an operating sensitivity of 0.770 on the ROC for each prediction window. An operating sensitivity of 0.770 was chosen in order to highlight the performance of the MLA at a sensitivity greater than the highest sensitivity attained by the MMSE comparator. As noted in Table 3, at the 24-month prediction window, the MMSE comparator achieves a peak sensitivity of 0.769. Therefore, we chose to set an operating sensitivity of 0.770 to compare the MLA against the peak performance of the MMSE comparator. The performance of the MLA showcases a strong ability to identify individuals with an increased likelihood of progression from MCI to AD, as well as those who will likely not progress from MCI to AD. Supported by a meta-analysis showcasing an MMSE cutoff of 26/27 to indicate MCI to AD progression risk [8], we chose the cutoff of 27 for displaying the MMSE classifier performance metrics in Table 3 and Supplementary Table S6. The MLA outperformed the MMSE classifier for identification of progression or non-progression from MCI to AD (i.e., differentiation between AD and non-AD classes) for all prediction time windows, with the AUROC value being significantly higher for the MLA than for the corresponding MMSE classifier in a given prediction window (*p* < 0.05) except at 12 and 18 months. Specificity, PPV, and accuracy were significantly higher for each MLA compared to the corresponding MMSE classifier in each prediction window except for the MLA at 42 months.

**Figure 4:**
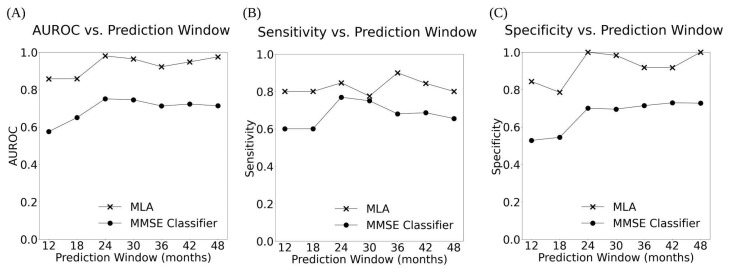
The AUROC (**A**), Sensitivity (**B**), and Specificity (**C**) vs. Prediction Window for the MLA and MMSE classifier across all 7 prediction windows showing the performance of the MLA and the MMSE over time.

Overall, the MLA, KNN model, MLP model, and LR model performed significantly better than the MMSE classifier at all prediction windows, for example as shown in Figure 3 and Figure 4 and Table 3 (as well as Supplementary Figures S2 and S4, and Supplementary Table S6). MMSE performance declined over time for all but two metrics (specificity and PPV, which both improved), whereas the MLA and MLP model improved on all but one metric (NPV) over time, the KNN improved on all metrics besides sensitivity and NPV, and the LR improved on all metrics besides NPV. The MLA largely performed similarly compared to the MLP and LR models, while the MLA performed significantly better than the KNN model at certain prediction windows in terms of specificity (at 12, 18, 42, and 48 months), PPV (at 42 and 48 months), and accuracy (at 12 and 18 months).

The 95% CIs for the MLA were consistently narrow, whereas the 95% CIs for the MMSE classifier were much broader (Table 3 and Supplementary Table S6), indicating the superior performance of the MLA. Additionally, there was little to no overlap between the 95% CIs for the AUROC and specificity for the MLA and the MMSE classifier, which further underlines the superiority of the MLA over the MMSE classifier.

**Figure 5:**
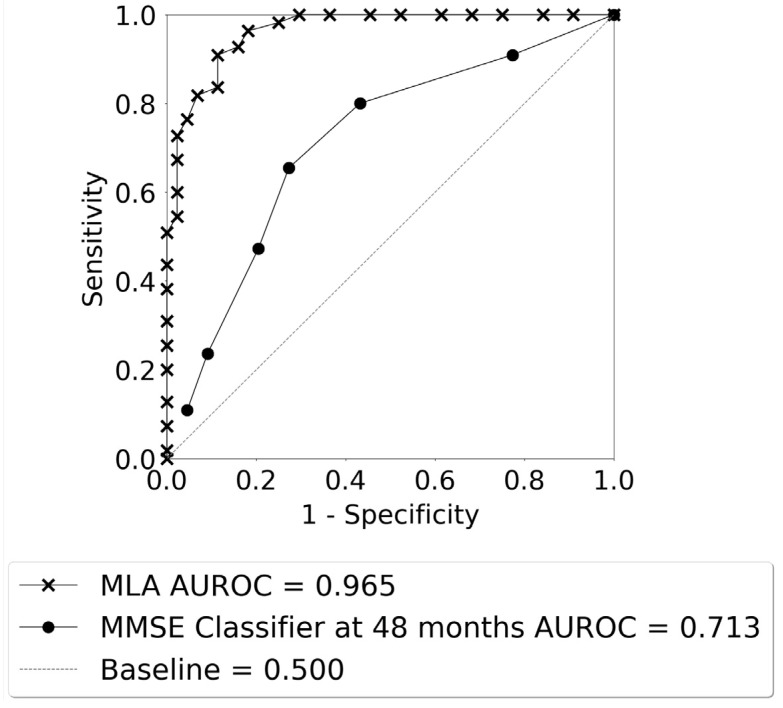
The ROC curves for AD class vs. non-AD class as given by the averaged MLA for the 24–48 months lookahead timeframe, and the MMSE classifier at 48 months.

Figure 5 displays the ROC curves for identification of progression vs. non-progression from MCI to AD for the averaged MLA. The AUROC for the MLA (0.965) was excellent and far superior to the performance of the MMSE classifier for longitudinal prediction at the 48-month prediction window (0.713).

The performance of the averaged MLA for differentiating between the AD class and the non-AD class is shown in Table 4 for the 24–48-month lookahead timeframe, with a bar plot visualization of these data shown in Supplementary Figure S5. The MLA performance metrics in Table 4 were obtained by selecting an operating sensitivity of 0.770 on the ROC. A cutoff of 27 was used for calculating the performance metrics for the MMSE classifier at the 48-month prediction window (Table 4). The averaged MLA outperformed the MMSE classifier for differentiation between the AD and non-AD classes.

The top 10 features impacting the performance of the MLA are shown in Table 5 for the 12-, 24-, and 48-month prediction windows. For the MLA at each prediction window, these ranked lists of most important features were determined using SHAP, a widely used approach to explaining the contribution of each feature towards the output (prediction) of a machine learning model [50]. The features are listed in the table from the most important feature at the top, decreasing in order of importance to the bottom. Similarly, the top 10 features for the 18-, 30-, 36-, and 42-month prediction windows are shown in Supplementary Table S7 and the individual features that were included for each neuropsychological assessment input are shown in Supplementary Table S5. Overall, for the windows comprising the averaged MLA, the 10 most important features for the prediction algorithm models were (from the most important to the least): Functional Assessment Questionnaire (FAQ) total score, Rey Auditory Verbal Learning Test (RAVLT) Immediate Recall subscore, ethnicity, 11-task Alzheimer’s Disease Assessment Scale–Cognitive Subscale (ADAS-CoG-11) total score, RAVLT Percent Forgetting, MMSE tree score, FAQ Tax Forms subscore, Logical memory Delayed Recall total score, 13-task ADAS-CoG (ADAS-Cog-13) Delayed Word Recall score, and FAQ Remembering subscore.

## 4. Discussion

Across six metrics, our MLA outperformed the SOC (MMSE), achieving significantly higher specificity and PPV for all prediction windows from 12 to 48 months, significantly higher accuracy for all but one prediction window, and significantly higher AUROC for all but two prediction windows. This consistent predictive improvement indicates that the MLA makes relatively stable gains in identifying patient progression from MCI to AD over time, in comparison to the SOC. Furthermore, it is suggested that longitudinal measurements can create a better estimation of how an individual’s brain and cognition are changing [41]. All four machine learning models (the XGBoost-based MLA and KNN, MLP, and LR comparison models) outperformed the SOC for all prediction windows, indicating that machine learning more broadly offers substantial value in predicting progression of MCI to AD over time. Further, the MLA performed significantly better than the KNN and LR models in certain key performance metrics, whereas it performed similarly to the more complex MLP model.

The KNN model, MLP model, and LR model were developed as additional comparators to further examine the potential of machine learning for this application. All three comparison models were able to reliably identify progression (or lack thereof) from MCI to AD over time with relatively high-performance metrics. In addition, the three comparison models outperformed the SOC comparator. However, there are some important limitations to the KNN, MLP, and LR approaches compared to the XGBoost-based MLA. First, the performance of the XGBoost-based MLA is significantly better than the KNN model at some time windows in terms of specificity (at 12, 18, 42, and 48 months), PPV (at 42 and 48 months), and accuracy (at 12 and 18 months). The XGBoost-based MLA also performed significantly better than the LR at the 12-month time window in terms of specificity and accuracy. Second, all three comparison models require additional data processing and filtering (or imputation) to enable their use for datasets containing missing data, which severely limits their application to clinical datasets such as those used here, which are often incomplete. While filtering was performed to eliminate features with high levels of missingness, the majority of patients in the total dataset had at least one missing feature. Without a robust imputation process or the ability to handle null values, many of these patients may not be eligible for prediction, reducing the clinical utility of this type of approach. In addition, imputation has limitations as generation of synthetic data may not be possible or ideal in clinical settings. Finally, MLP is a neural network, a category of machine learning approaches which are notorious black boxes, in the sense that they are extremely challenging to interpret. As feature importance is a fundamental element of applied machine learning, particularly for clinical algorithms which can be used to guide medical treatment, an approach such as XGBoost is preferable given its relatively high level of interpretability.

In our work, we endeavored to develop and validate an ML-based tool to provide a longitudinal prediction regarding the risk of progressing to AD in patients with an existing MCI diagnosis. It is the intent for such a tool to facilitate an easier risk assessment process for HCPs using highly accessible and personalized patient data and with better accuracy than existing screening tools. The patient data used is pre-existing and readily available data from the EHR, as patients who are diagnosed with MCI (those for whom the MLA is intended for use) undergo comprehensive assessments [52]. Traditionally, assessing a patient’s risk of progressing from an MCI diagnosis to AD is a complex, multi-step process that includes the use of SOC risk assessment scales, ongoing and repeated evaluation to measure changes in an individual’s cognitive status, and clinical evaluation to rule out other potential causes of brain disease or cognitive impairment. HCPs have a range of assessments available to select from and may need to tailor their selection based on the patient’s symptoms. Additionally, a variety of assessments that examine different symptoms, domains, and levels of functioning may have to be used [12,53,54]. The most commonly used SOC risk assessment scales, MoCA and MMSE, have been validated for determining the rate at which an individual with MCI will experience cognitive decline, though they were not originally created or intended for use to capture data over time. Possibly as a result of not being designed for such use, the MMSE and MoCA have been reported to lose sensitivity when measuring cognitive decline over time [41]. However, even with serial measurements taken over time, changes to an individual’s cognition are not always obvious [8], which makes estimation of the risk of progression challenging. Cognitive changes during the prodromal stage of AD, when AD-associated MCI may be present, may be even less obvious than symptoms at a more advanced stage of AD, and thus harder to attribute to future AD onset. AD biomarkers in patients with MCI may suggest a risk of progression from MCI to AD; however, the invasive and costly nature of such tests may make accessibility difficult and require specialized settings and equipment. In contrast, our MLA achieved excellent performance for making predictions within 12-, 18-, 24-, 30-, 36-, 42-, or 48-month prediction windows after baseline (highest AUROC: 0.980 for prediction at 24 months) and with the longitudinal data averaged (AUROC: 0.965). All metrics were significantly more robust than the MMSE SOC used for prediction on the same cohort. Most impressively, the MLA at the 36-month prediction window achieved values ≥ 0.900 for all six performance metrics (AUROC of 0.922, sensitivity of 0.900, specificity of 0.918, PPV of 0.918, NPV of 0.900, and accuracy of 0.909), with each performance measure significantly higher than the corresponding measure for the MMSE classifier (AUROC of 0.712, sensitivity of 0.680, specificity of 0.714, PPV of 0.708, NPV of 0.686, and accuracy of 0.697). Because the MLA draws data directly from EHRs and uses individualized patient data, it could be used in a primary care setting by an HCP without specialized expertise, and with better performance than the MMSE SOC. Research on predictive tools using readily available patient data to determine the risk of converting from MCI to AD is limited; thus, this approach fills an unmet clinical need. Grassi et al. used ML models and sociodemographic data, results from neuropsychological tests, and MCI categorization (early or late) to predict progression from MCI to AD within 3 years after MCI diagnosis, with predictions demonstrating performance values of 0.88 for AUROC, >0.77 for sensitivity, and ≈0.80 for specificity [55]. Bucholc et al. employed an unsupervised MLA to predict conversion from MCI to dementia 4 years after MCI diagnosis using data from cognitive and daily functioning assessments, with the best performing model achieving an accuracy of 0.875 [56].

A meta-analysis of examining the MMSE noted highly variable performance for estimating conversion from MCI to AD within varying lookahead periods, between 15 months and 7 years (sensitivity: 27–89%; specificity: 32–90%) [8]. MMSE also has limitations that may render its use less suitable for subpopulations of individuals. For example, it is well-documented in literature that the MMSE scores are subject to the “ceiling and floor effects” [10,41]. In these particular circumstances, individuals in a pre-dementia stage can achieve MMSE scores indicating normal cognition; for individuals who have severe cognitive impairment, the test loses sensitivity to evaluate ongoing decline. Socio-demographic factors, such as age, level of formal education, race, and ethnicity also impact how an individual scores on the MMSE, thus making it necessary for an HCP to consider those factors when selecting thresholds for interpreting the scores. MMSE has multiple thresholds by which a clinician could determine that a patient has a positive MMSE result; for example, it is suggested that individuals with higher levels of education should be scored within a higher threshold, and vice versa for individuals that have received less education (equating to a higher number of points achieved on the MMSE) to avoid the ceiling and floor effects, respectively. MoCA is also subject to similar limitations in that it was not intended for longitudinal use to repeatedly collect data for the purpose of measuring cognitive decline over time and a different threshold must be used based on the individual’s level of education to correctly interpret scores for determining the rate of cognitive decline. Though there are limited studies validating MoCA for identification of patients with MCI who are at high risk of progressing to dementia, the studies that were conducted report AUROCs ranging from 0.740 to 0.95 [57,58].

In comparison to the MMSE SOC, a multi-parameter model utilizing various relevant demographic and assessment data, including neuropsychiatric assessments, is likely to be more robust to longitudinal and practice effects, as it incorporates several standardized assessments that are known to continue working relatively effectively over time. This extends to the RAVLT and logical memory tests. Combined with a significantly enhanced predictive power versus the SOC, this careful inclusion of diverse information sources may ensure more reliable application for clinical use. Averaging MLA predictions over time may also improve the prediction quality, especially when predictions for a given individual may vary over time.

### Limitations and Future Directions

Though this proof-of-concept study demonstrates promise that ML methods hold the ability to provide accurate and automated risk stratification for progression from MCI to AD, our study has several limitations. The dataset that we used contained retrospective patient data. To determine the MLA’s effectiveness in a clinical setting, and particularly in a non-specialized setting (e.g., primary care), future work should deploy the MLA for validation using prospective patient data. While the ADNI dataset is an invaluable tool for AD research, data were collected solely from clinical sites in the US and Canada [35]. Therefore, this does not take into account regional and population-specific variations, both in terms of progression risk assessment and diagnostic thresholds for MCI and AD and global prevalence estimates [12]. Future work should consider stratifying individuals by racial, ethnic, and geographic demographics and training and testing the MLA on these subgroups to ensure that the MLA achieves similar performance metrics across diverse populations in order to reduce demographic bias and encourage health equity. Related to the demographic variable of sex, several limitations exist. Though females have a higher prevalence of AD than males [59], the demographics within our study population do not reflect this, resulting in imbalanced data. There are notable differences among sexes between domains within neuropsychological screening tools in terms of how they contribute to determining risk of progression from MCI to AD [60]. Females also experience different rates of conversion from MCI to AD than males [60]. To ensure that our MLA can provide the most accurate prediction of progression in these two cohorts, future research should stratify patients by sex for separate training and validation. Regarding our second experiment, in which data from the lookahead time windows were averaged, there is a lack of comparable studies regarding these averaging methods. However, prediction within this broad timeframe (12–48 months) may enhance the clinical utility and ease of use of the MLA for predicting MCI progression to AD. Therefore, future work should provide further validation of these statistical methods.

## 5. Conclusions

The current limitations associated with the evaluation of individuals with MCI for risk of progression to AD, including interpretation of biomarker thresholds and the high cost of invasive testing, result in a deficient screening process. Though SOC tools are useful in a variety of clinical settings, they must be used in conjunction with comprehensive clinical assessments, and accuracy may be limited in different subpopulations. ML overcomes these limitations through the use of personalized data that are collected automatically from EHRs. It has the potential to provide broader access to risk assessment screening with higher accuracy than current methods, which may reduce costs associated with unnecessary invasive testing, allow for better care planning for individuals at risk of AD, and provide the opportunity for individuals who are not at risk for AD to seek alternative diagnoses and treatment to manage cognitive decline. In this study, using a comprehensive ML approach led to significant improvement across many predictive performance measures, compared to the SOC, without requiring new or more complex testing of patients, where averaging of predictions over time yielded a more stable prediction of progression from MCI to Alzheimer’s disease. Further testing, evaluation, and application of this approach in clinical settings will afford clinicians a reliable tool when evaluating the risk of cognitive decline.

## Figures and Tables

**Table 1:** Demographic information for training sets, for three timepoints.

		12 Months		24 Months		48 Months	
Demographics (Training Sets)		Non-AD (*n*= 338)	AD (*n* = 56)	Non-AD (*n* = 257)	AD (*n* = 137)	Non-AD (*n* = 190)	AD (*n* = 204)
Age (years)	55–60	9 (2.7%)	4 (7.1%)	8 (3.1%)	5 (3.6%)	7 (3.7%)	6 (2.9%)
	61–70	108 (32.0%)	10 (17.9%)	91 (35.4%)	27 (19.7%)	73 (38.4%)	45 (22.1%)
	71–80	160 (47.3%)	32 (57.1%)	115 (44.7%)	77 (56.2%)	87 (45.8%)	105 (51.5%)
	81–90	61 (18.0%)	10 (17.9%)	43 (16.7%)	28 (20.4%)	23 (12.1%)	48 (23.5%)
Sex Assigned at Birth	Female	128 (37.9%)	26 (46.4%)	100 (38.9%)	54 (39.4%)	71 (37.4%)	83 (40.7%)
	Male	210 (62.1%)	30 (53.6%)	157 (61.1%)	83 (60.6%)	119 (62.6%)	121 (59.3%)
Race	White	314 (92.9%)	54 (96.4%)	238 (92.6%)	130 (94.9%)	178 (93.7%)	190 (93.1%)
	Black or African American	11 (3.3%)	1 (1.8%)	9 (3.5%)	3 (2.2%)	5 (2.6%)	7 (3.4%)
	Asian	8 (2.4%)	1 (1.8%)	6 (2.3%)	3 (2.2%)	3 (1.6%)	6 (2.9%)
	American Indian or Alaskan Native	1 (0.2%)	0 (0.0%)	1 (0.4%)	0 (0.0%)	1 (0.5%)	0 (0.0%)
	More than one race	4 (1.2%)	0 (0.0%)	3 (1.2%)	1 (0.7%)	3 (1.6%)	1 (0.5%)
Ethnicity	Hispanic/Latino	11 (3.3%)	1 (1.8%)	9 (3.5%)	3 (2.2%)	6 (3.2%)	6 (2.9%)
	Not Hispanic/Latino	327 (96.7%)	55 (98.2%)	248 (96.5%)	134 (97.8%)	184 (96.8%)	198 (97.1%)
Comorbidities	Diabetes	28 (8.3%)	6 (10.7%)	24 (9.3%)	10 (7.3%)	18 (9.5%)	16 (7.8%)
	Depression	109 (32.2%)	14 (25.0%)	84 (32.7%)	39 (28.5%)	59 (31.1%)	64 (31.4%)
	Osteoporosis or Osteoarthritis	81 (24.0%)	11 (19.6%)	62 (24.1%)	30 (21.9%)	45 (23.7%)	47 (23.0%)
	Cerebrovascular Disease	15 (4.4%)	2 (3.6%)	13 (5.1%)	4 (2.9%)	8 (4.2%)	9 (4.4%)
	Hypertension	141 (41.7%)	27 (48.2%)	108 (42.0%)	60 (43.8%)	81 (42.6%)	87 (42.6%)
	Hearing or vision impairment	85 (25.1%)	10 (17.9%)	63 (24.5%)	32 (23.4%)	47 (24.7%)	48 (23.5%)
	Coronary heart disease	16 (4.7%)	1 (1.8%)	15 (5.8%)	2 (1.5%)	10 (5.3%)	7 (3.4%)

**Table 2:** MLA inputs used in feature selection for all MLA models and timepoints.

**Demographics** Age Marital Status	**Neuropsychiatric Assessments** Alzheimer’s Disease Assessment Scale (ADAS) Mini-Mental State Examination (MMSE) Functional Activities Questionnaire (FAQ) Neuropsychiatric Inventory Questionnaire (NPI-Q) Clinical Dementia Rating (CDR) Geriatric Depression Scale (GDS) Rey Auditory Verbal Learning Test (RAVLT) Neuropsychological Assessment Battery (NAB) ADNI-specific composite scoring Modified Hachinski Ischemic Score
**Family Medical History**	
**Comorbidities**	

**Table 3:** MLA performance on corresponding hold-out test sets for 12-, 24-, and 48-month prediction windows showing the performance of the MLA, in comparison to the MMSE and three comparison models over time.

		Performance Metrics					
Prediction Window	Modeling Approach	AUROC (95% CI)	Sensitivity (95% CI)	Specificity (95% CI)	PPV (95% CI)	NPV (95% CI)	Accuracy (95% CI)
12 months	MLA	0.857 (0.756–0.935)	0.800 (0.646–0.954)	0.843 (0.796–0.890)	0.364 (0.239–0.489)	0.974 (0.952–0.996)	0.838 (0.793–0.883)
	LR	0.740 (0.589–0.877)	0.800 (0.783–0.817)	0.686 (0.678–0.693)	0.267 (0.256–0.278)	0.960 (0.956–0.964)	0.700 (0.693–0.707)
	MLP	0.829 (0.719–0.934)	0.800 (0.784–0.816)	0.771 (0.765–0.778)	0.364 (0.239–0.489)	0.974 (0.952–0.996)	0.838 (0.793–0.883)
	KNN	0.789 (0.635–0.921)	0.900 (0.863–0.937)	0.557 (0.534–0.580)	0.225 (0.200–0.250)	0.975 (0.965–0.985)	0.600 (0.579–0.621)
	MMSE Classifier ^⧧^	0.575 (0.334–0.804)	0.600 (0.270–0.930)	0.528 (0.415–0.641)	0.125 (0.023–0.227)	0.922 (0.841–1.000)	0.535 (0.429–0.642)
24 months	MLA	0.980 (0.957–0.996)	0.846 (0.762–0.931)	1.000 (0.998–1.000)	1.000 (0.998–1.000)	0.909 (0.857–0.961)	0.939 (0.904–0.975)
	LR	0.957 (0.910–0.988)	0.816 (0.727–0.904)	0.952 (0.906–0.999)	0.939 (0.881–0.998)	0.851 (0.778–0.924)	0.888 (0.838–0.937)
	MLP	0.960 (0.909–0.991)	0.789 (0.682–0.897)	1.000 (0.998–1.000)	1.000 (0.998–1.000)	0.840 (0.756–0.924)	0.900 (0.846–0.954)
	KNN	0.975 (0.943–0.996)	0.789 (0.700–0.879)	0.976 (0.944–1.000)	0.968 (0.925–1.000)	0.837 (0.765–0.908)	0.888 (0.840–0.935)
	MMSE Classifier ^⧧^	0.750 (0.642–0.848)	0.769 (0.626–0.913)	0.700 (0.574–0.826)	0.625 (0.476–0.774)	0.824 (0.710–0.937)	0.727 (0.632–0.823)
48 months	MLA	0.975 (0.947–0.995)	0.800 (0.715–0.885)	1.000 (0.998–1.000)	1.000 (0.998–1.000)	0.800 (0.715–0.885)	0.889 (0.839–0.938)
	LR	0.964 (0.919–0.994)	0.816 (0.724–0.908)	0.976 (0.942–1.000)	0.969 (0.924–1.000)	0.854 (0.780–0.929)	0.900 (0.851–0.949)
	MLP	0.965 (0.920–0.995)	0.895 (0.814–0.976)	0.976 (0.938–1.000)	0.971 (0.926–1.000)	0.911 (0.842–0.980)	0.938 (0.893–0.982)
	KNN	0.944 (0.890–0.979)	0.789 (0.711–0.868)	0.952 (0.913–0.992)	0.938 (0.887–0.988)	0.833 (0.769–0.897)	0.875 (0.831–0.919)
	MMSE Classifier ^⧧^	0.713 (0.601–0.813)	0.655 (0.518–0.791)	0.727 (0.584–0.870)	0.750 (0.617–0.883)	0.627 (0.483–0.772)	0.687 (0.588–0.786)

^⧧^ Note: The MMSE classifier uses a threshold score value of 27. AUROC = area under the receiver operator characteristic curve; CI = confidence interval; KNN = *k*-nearest neighbors algorithm; MLP = multi-layer perceptron; PPV = positive predictive value; NPV = negative predictive value.

**Table 4:** Averaged MLA performance in comparison with the performance of the MMSE classifier at 48 months.

Performance Metrics	Averaged MLA	MMSE Classifier at 48 Months ^⧧^
AUROC (95% CI)	0.965 (0.927–0.990)	0.713 (0.601–0.813)
Sensitivity (95% CI)	0.800 (0.734–0.866)	0.655 (0.518–0.791)
Specificity (95% CI)	0.955 (0.915–0.993)	0.727 (0.584–0.870)
PPV (95% CI)	0.957 (0.920–0.993)	0.750 (0.617–0.883)
NPV (95% CI)	0.792 (0.724–0.861)	0.627 (0.483–0.772)
Accuracy (95% CI)	0.869 (0.827–0.910)	0.687 (0.688–0.786)

^⧧^ Note: The MMSE classifier uses a threshold score value of 27. AUROC = area under the receiver operator characteristic curve; CI = confidence interval; PPV = positive predictive value; NPV = negative predictive value.

**Table 5:** The top 10 features impacting the performance of the MLA at 12-, 30-, and 48-month prediction windows.

12 Months	24 Months	48 Months
FAQ Game subscore MMSE day of the week CDR sum of boxes MMSE tree FAQ Attention and Understanding subscore MMSE flag Ethnicity FAQ Remembering subscore RAVLT Percent Forgetting MMSE letter “L” in “spell WORLD backwards” task	11. RAVLT Immediate Recall subscore 12. Ethnicity 13. RAVLT Percent Forgetting 14. Logical Memory Delayed Recall total score 15. ADAS-Cog-11 Orientation 16. MMSE tree 17. MMSE day of the week 18. FAQ total score 19. GDS total score 20. ADAS-Cog-13 Delayed Word Recall	21. Logical Memory Delayed Recall total score 22. Ethnicity 23. ADAS-Cog-13 Delayed Word Recall 24. FAQ Travel subscore 25. RAVLT Immediate Recall subscore 26. FAQ total score 27. ADAS-Cog-13 Number Cancellation 28. RAVLT Learning subscore 29. FAQ Tax Forms subscore 30. NPIQ time to complete

FAQ = Functional Assessment Questionnaire; MMSE = Mini-Mental State Examination; CDR = Clinical Dementia Rating; RAVLT = Rey Auditory Verbal Learning Test; ADAS-Cog-11 = Alzheimer’s Disease Assessment Scale–Cognitive Subscale (11 tasks); GDS = Geriatric Depression Scale; ADAS-Cog-13 = Alzheimer’s Disease Assessment Scale–Cognitive Subscale (13 tasks); NPIQ = Neuropsychiatric Inventory Questionnaire.

## Data Availability

The data used for analysis in this paper, Alzheimer’s Disease Neuroimaging Initiative (ADNI), are available from a publicly available database.

## Supplementary Materials

The following supporting information can be downloaded at: https://www.mdpi.com/article/10.3390/diagnostics14010013/s1. Table S1. Machine learning algorithm (MLA) comparison with other mild cognitive impairment (MCI) and Alzheimer’s disease (AD) classifiers. Table S2. Demographic information for training sets, for months 18, 30, 36, and 42 after baseline. Table S3. Demographic information for hold-out test sets, for months 12, 24, and 48 after baseline. Table S4. Demographic information for hold-out test sets, for months 18, 30, 36, and 42 after baseline. Figure S1. The receiver operating characteristic (ROC) curves for the Alzheimer’s disease (AD) class vs. the non-AD class as given by the Mini-Mental State Examination (MMSE) classifier when the prediction algorithm is tested on the hold-out test sets, for each of the seven prediction windows showing performance of the machine learning algorithm (MLA) and the MMSE over time. Figure S2. The receiver operating characteristic (ROC) curves for the Alzheimer’s disease (AD) class vs. the non-AD class as given by the machine learning algorithm (MLA) and the Mini-Mental State Examination (MMSE) classifier showing performance of the MLA and the MMSE over time: (A) 18-month prediction window; (B) 30-month prediction window; (C) 36-month prediction window; (D) 42-month prediction window. Figure S3. The receiver operating characteristic (ROC) curves for the Alzheimer’s disease (AD) class vs. the non-AD class as given by the machine learning algorithm (MLA) classifier when the prediction algorithm is tested on the hold-out test sets for each of the seven prediction windows showing performance of the MLA over time. Figure S4. The positive predictive value (PPV) (A), negative predictive value (NPV) (B), and accuracy (C) vs. prediction window for the machine learning algorithm (MLA) and Mini-Mental State Examination (MMSE) classifiers across all seven prediction windows showing performance of the MLA and the MMSE over time. Table S5. Detailed feature inputs for all input categories. Table S6. Machine learning algorithm (MLA) performance on corresponding hold-out test sets for the 18-, 30-, 36-, and 42-month prediction windows showing performance of the XGBoost-based MLA, the multi-layer perceptron (MLP) neural network, *k*-nearest neighbors (KNN), and logistic regression (LR) models, and the Mini-Mental State Examination (MMSE) over time. XGBoost is a widely used gradient-boosted tree ensemble method. Figure S5. Bar plot visualization of averaged machine learning algorithm (MLA) performance in comparison with the performance of the Mini-Mental State Examination (MMSE) classifier at 48 months. Each pair of bars represents a different performance metric: area under the receiver operating characteristic curve (AUROC), sensitivity, specificity, positive predictive value (PPV), negative predictive value (NPV), and accuracy. The value on each bar represents the calculated metric for either the MLA or MMSE classifier, and the error bars at the top of each bar represent 95% confidence intervals. Table S7. The top 10 features impacting the performance of the machine learning algorithm (MLA) at the 18-, 30-, 36-, and 42-month prediction windows.

## References

[1] Livingston G., Huntley J., Sommerlad A., Ames D., Ballard C., Banerjee S., Brayne C., Burns A., Cohen-Mansfield J., Cooper C. (2020). Dementia prevention, intervention, and care: 2020 report of the Lancet Commission. Lancet Lond. Engl..

[2] Riffin C., Van Ness P.H., Wolff J.L., Fried T. (2019). A Multifactorial Examination of Caregiver Burden in a National Sample of Family and Unpaid Caregivers. J. Am. Geriatr. Soc..

[3] Alzheimer’s Association 2022 Alzheimer’s Disease Facts & Figures Special Report. Alzheimers Dement 2022; 18. 2022-Facts-and-Figures-Report_1.pdf (). http://www.alz.org.

[4] Skaria A.P. (2022). The economic and societal burden of Alzheimer disease: Managed care considerations. Am. J. Manag. Care.

[5] Busse A., Angermeyer M.C., Riedel-Heller S.G. (2006). Progression of mild cognitive impairment to dementia: A challenge to current thinking. Br. J. Psychiatry.

[6] Muñoz-Bermejo L., González-Becerra M.J., Barrios-Fernández S., Postigo-Mota S., Jerez-Barroso M.D., Martínez J.A., Suárez-Lantarón B., Marín D.M., Martín-Bermúdez N., Ortés-Gómez R. (2022). Cost-Effectiveness of the Comprehensive Interdisciplinary Program-Care in Informal Caregivers of People with Alzheimer’s Disease. Int. J. Environ. Res. Public Health.

[7] Hurd M.D., Martorell P., Delavande A., Mullen K.J., Langa K.M. (2013). Monetary Costs of Dementia in the United States. N. Engl. J. Med..

[8] Arevalo-Rodriguez I., Smailagic N., Roqué IFiguls M., Ciapponi A., Sanchez-Perez E., Giannakou A., Pedraza O.L., Cosp X.B., Cullum S. (2015). Mini-Mental State Examination (MMSE) for the detection of Alzheimer’s disease and other dementias in people with mild cognitive impairment (MCI). Cochrane Database Syst. Rev..

[9] Parnetti L., Chipi E., Salvadori N., D’Andrea K., Eusebi P. (2019). Prevalence and risk of progression of preclinical Alzheimer’s disease stages: A systematic review and meta-analysis. Alzheimers Res. Ther..

[10] Jia X., Wang Z., Huang F., Su C., Du W., Jiang H., Wang H., Wang J., Wang F., Su W. (2021). A comparison of the Mini-Mental State Examination (MMSE) with the Montreal Cognitive Assessment (MoCA) for mild cognitive impairment screening in Chinese middle-aged and older population: A cross-sectional study. BMC Psychiatry.

[11] Assessing Risk for Alzheimer’s Disease Natl Inst Aging n.d. https://www.nia.nih.gov/health/assessing-risk-alzheimers-disease.

[12] Tahami Monfared A.A., Byrnes M.J., White L.A., Zhang Q. (2022). Alzheimer’s Disease: Epidemiology and Clinical Progression. Neurol. Ther..

[13] Clark D.G., McLaughlin P.M., Woo E., Hwang K., Hurtz S., Ramirez L., Eastman J., Dukes R.M., Kapur P., DeRamus T.P. (2016). Novel verbal fluency scores and structural brain imaging for prediction of cognitive outcome in mild cognitive impairment. Alzheimers Dement. Diagn. Assess. Dis. Monit..

[14] Burdick H., Pino E., Gabel-Comeau D., Gu C., Roberts J., Le S., Slote J., Saber N., Pellegrini E., Green-Saxena A. (2020). Validation of a machine learning algorithm for early severe sepsis prediction: A retrospective study predicting severe sepsis up to 48 h in advance using a diverse dataset from 461US hospitals. BMC Med. Inform. Decis. Mak..

[15] Fisher C.K., Smith A.M., Walsh J.R. (2019). Machine learning for comprehensive forecasting of Alzheimer’s Disease progression. Sci. Rep..

[16] Garikipati A., Ciobanu M., Singh N.P., Barnes G., Decurzio J., Mao Q., Das R. (2023). Clinical Outcomes of a Hybrid Model Approach to Applied Behavioral Analysis Treatment. Cureus.

[17] Hinrichs C., Singh V., Xu G., Johnson S.C. (2011). Predictive markers for AD in a multi-modality framework: An analysis of MCI progression in the ADNI population. NeuroImage.

[18] Le S., Allen A., Calvert J., Palevsky P.M., Braden G., Patel S., Pellegrini E., Green-Saxena A., Hoffman J., Das R. (2021). Convolutional Neural Network Model for Intensive Care Unit Acute Kidney Injury Prediction. Kidney Int. Rep..

[19] Maharjan J., Garikipati A., Dinenno F.A., Ciobanu M., Barnes G., Browning E., DeCurzio J., Mao Q., Das R. (2023). Machine learning determination of applied behavioral analysis treatment plan type. Brain Inform..

[20] Massetti N., Russo M., Franciotti R., Nardini D., Mandolini G.M., Granzotto A., Bomba M., Delli Pizzi S., Mosca A., Scherer R. (2022). A Machine Learning-Based Holistic Approach to Predict the Clinical Course of Patients within the Alzheimer’s Disease Spectrum 1. J. Alzheimers Dis..

[21] Mohamadlou H., Panchavati S., Calvert J., Lynn-Palevsky A., Le S., Allen A., Pellegrini E., Green-Saxena A., Barton C., Fletcher G. (2020). Multicenter validation of a machine-learning algorithm for 48-h all-cause mortality prediction. Health Inform. J..

[22] Ryan L., Mataraso S., Siefkas A., Pellegrini E., Barnes G., Green-Saxena A., Hoffman J., Calvert J., Das R. (2021). A Machine Learning Approach to Predict Deep Venous Thrombosis Among Hospitalized Patients. Clin. Appl. Thromb. Hemost..

[23] Thapa R., Garikipati A., Ciobanu M., Singh N., Browning E., DeCurzio J., Barnes G., Dinenno F.A., Mao Q., Das R. (2023). Machine Learning Differentiation of Autism Spectrum Sub-Classifications. J. Autism Dev. Disord..

[24] Thapa R., Iqbal Z., Garikipati A., Siefkas A., Hoffman J., Mao Q., Das R. (2022). Early prediction of severe acute pancreatitis using machine learning. Pancreatology.

[25] Morabito F.C., Campolo M., Labate D., Morabito G., Bonanno L., Bramanti A., De Salvo S., Marra A., Bramanti P. (2015). A Longitudinal EEG Study of Alzheimer’s Disease Progression Based on A Complex Network Approach. Int. J. Neural Syst..

[26] Orovas C., Orovas E., Dagla M., Daponte A., Rigas N., Ougiaroglou S., Iatrakis G., Antoniou E. (2022). Neural Networks for Early Diagnosis of Postpartum PTSD in Women after Cesarean Section. Appl. Sci..

[27] Mallo S.C., Valladares-Rodriguez S., Facal D., Lojo-Seoane C., Fernández-Iglesias M.J., Pereiro A.X. (2020). Neuropsychiatric symptoms as predictors of conversion from MCI to dementia: A machine learning approach. Int. Psychogeriatr..

[28] Rutkowski T.M., Abe M.S., Komendzinski T., Sugimoto H., Narebski S., Otake-Matsuura M. (2023). Machine learning approach for early onset dementia neurobiomarker using EEG network topology features. Front. Hum. Neurosci..

[29] Spasov S., Passamonti L., Duggento A., Liò P., Toschi N. (2019). A parameter-efficient deep learning approach to predictconversion from mild cognitive impairment to Alzheimer’s disease. NeuroImage.

[30] Marcisz A., Polanska J. (2023). Can T1-Weighted Magnetic Resonance Imaging Significantly Improve Mini-Mental State Examination-Based Distinguishing Between Mild Cognitive Impairment and Early-Stage Alzheimer’s Disease?. J. Alzheimers Dis..

[31] Peng J., Wang W., Qiaowei S., Jie H., Hui J., Xue Q., Zhongyu Y., Yuguo W., Zhenyu S. (2023). 18F-FDG-PET Radiomics Based on White Matter Predicts The Progression of Mild Cognitive Impairment to Alzheimer Disease: A Machine Learning Study. Acad. Radiol..

[32] Chen Z., Liu Y., Zhang Y., Li Q. (2023). Alzheimer’s Disease Neuroimaging Initiative. Orthogonal latent space learning with feature weighting and graph learning for multimodal Alzheimer’s disease diagnosis. Med. Image Anal..

[33] Martínez-Torteya A., Treviño V., Tamez-Peña J.G. (2015). Improved Diagnostic Multimodal Biomarkers for Alzheimer’s Disease and Mild Cognitive Impairment. Biomed. Res. Int..

[34] Chen T., Guestrin C. XGBoost: A Scalable Tree Boosting System. Proceedings of the 22nd ACM SIGKDD International Conference on Knowledge Discovery and Data Mining.

[35] ADNI|Alzheimer’s Disease Neuroimaging Initiative n.d. https://adni.loni.usc.edu/.

[36] Petersen R.C., Aisen P.S., Beckett L.A., Donohue M.C., Gamst A.C., Harvey D.J., Jack C.R., Jagust W.J., Shaw L.M., Toga A.W. (2010). Alzheimer’s Disease Neuroimaging Initiative (ADNI). Neurology.

[37] Ikram M., Innes K., Sambamoorthi U. (2019). Association of osteoarthritis and pain with Alzheimer’s Diseases and Related Dementias among older adults in the United States. Osteoarthr. Cartil..

[38] Murphy C. (2019). Olfactory and other sensory impairments in Alzheimer disease. Nat. Rev. Neurol..

[39] Santiago J.A., Potashkin J.A. (2021). The Impact of Disease Comorbidities in Alzheimer’s Disease. Front. Aging Neurosci..

[40] Shwartz-Ziv R., Armon A. (2022). Tabular data: Deep learning is not all you need. Inf. Fusion..

[41] Mendez M.F. *Up to Date*. Mental Status Scales to Evaluate Cognition. https://www.uptodate.com/contents/mental-status-scales-to-evaluate-cognition#!.UpToDate2023.

[42] Uddin S., Haque I., Lu H., Moni M.A., Gide E. (2022). Comparative performance analysis of K-nearest neighbour (KNN) algorithm and its different variants for disease prediction. Sci. Rep..

[43] Rana A., Rawat A.S., Bijalwan A., Bahuguna H. Application of Multi Layer (Perceptron) Artificial Neural Network in the Diagnosis System: A Systematic Review. Proceedings of the 2018 International Conference on Research in Intelligent and Computing in Engineering (RICE).

[44] Levy J.J., O’Malley A.J. (2020). Don’t dismiss logistic regression: The case for sensible extraction of interactions in the era of machine learning. BMC Med. Res. Methodol..

[45] Herrin J., Abraham N.S., Yao X., Noseworthy P.A., Inselman J., Shah N.D., Ngufor C. (2021). Comparative Effectiveness of Machine Learning Approaches for Predicting Gastrointestinal Bleeds in Patients Receiving Antithrombotic Treatment. JAMA Netw. Open.

[46] Xu Q., Lu X. (2022). Development and validation of an XGBoost model to predict 5-year survival in elderly patients with intrahepatic cholangiocarcinoma after surgery: A SEER-based study. J. Gastrointest. Oncol..

[47] Welcome to Python.org. PythonOrg 2023. https://www.python.org/.

[48] NumPy n.d. https://numpy.org/.

[49] Matplotlib—Visualization with Python n.d. https://matplotlib.org/.

[50] Scikit-Learn. Scikit-Learn n.d. https://scikit-learn/stable/about.html.

[51] Lundberg S.M., Erion G., Chen H., DeGrave A., Prutkin J.M., Nair B., Katz R., Himmelfarb J., Bansal N., Lee S.I. (2020). From local explanations to global understanding with explainable AI for trees. Nat. Mach. Intell..

[52] Petersen R.C., Lopez O., Armstrong M.J., Getchius T.S.D., Ganguli M., Gloss D., Gronseth G.S., Marson D., Pringsheim T., Day G.S. (2018). Practice guideline update summary: Mild cognitive impairment: Report of the Guideline Development, Dissemination, and Implementation Subcommittee of the American Academy of Neurology. Neurology.

[53] Goodarzi Z., Ismail Z. Neuropsychiatric Aspects of Alzheimer’s Disease. Pract Neurol 2019. https://practicalneurology.com/articles/2019-june/neuropsychiatric-aspects-of-alzheimers-disease.

[54] Cognitive Assessment Tools. Alzheimers Dis Dement n.d. https://alz.org/professionals/health-systems-medical-professionals/clinical-resources/cognitive-assessment-tools.

[55] Grassi M., Rouleaux N., Caldirola D., Loewenstein D., Schruers K., Perna G., Dumontier M., Alzheimer’s Disease Neuroimaging Initiative (2019). A Novel Ensemble-Based Machine Learning Algorithm to Predict the Conversion From Mild Cognitive Impairment to Alzheimer’s Disease Using Socio-Demographic Characteristics, Clinical Information, and Neuropsychological Measures. Front. Neurol..

[56] Bucholc M., Titarenko S., Ding X., Canavan C., Chen T. (2023). A hybrid machine learning approach for prediction of conversion from mild cognitive impairment to dementia. Expert. Syst. Appl..

[57] Julayanont P., Brousseau M., Chertkow H., Phillips N., Nasreddine Z.S. (2014). Montreal Cognitive Assessment Memory Index Score (MoCA-MIS) as a predictor of conversion from mild cognitive impairment to Alzheimer’s disease. J. Am. Geriatr. Soc..

[58] Liu X., Chen X., Zhou X., Shang Y., Xu F., Zhang J., He J., Zhao F., Du B., Wang X. (2021). Validity of the MemTrax Memory Test Compared to the Montreal Cognitive Assessment in the Detection of Mild Cognitive Impairment and Dementia due to Alzheimer’s Disease in a Chinese Cohort. J. Alzheimers Dis..

[59] Lin K.A., Choudhury K.R., Rathakrishnan B.G., Marks D.M., Petrella J.R., Doraiswamy P.M. (2015). Marked gender differences in progression of mild cognitive impairment over 8 years. Alzheimers Dement. Transl. Res. Clin. Interv..

[60] Berezuk C., Khan M., Callahan B.L., Ramirez J., Black S.E., Zakzanis K.K., Alzheimer’s Disease Neuroimaging Initiative (2023). Sex differences in risk factors that predict progression from mild cognitive impairment to Alzheimer’s dementia. J. Int. Neuropsychol. Soc..

