# Supplementary material for: Machine Learning Approach for Improved Longitudinal Prediction of Progression from Mild Cognitive Impairment to Alzheimer’s Disease
Source: Diagnostics (Basel). 2023 Dec 20;14(1):13. doi: 10.3390/diagnostics14010013 (PMC10795823; doi:10.3390/diagnostics14010013)
Supplement: Supplementary file 1 [file diagnostics-14-00013-s001.zip › diagnostics-2713839-Supplementary.pdf]

Table S1. Machine learning algorithm (MLA) comparison with other mild cognitive impairment (MCI) and Alzheimer's disease (AD) classifiers.

**Classifier Tool**	**Classification Task**	**Inputs**	**Performance Metrics**
MLA (XGBoost, this research) Adelson *et al.*	Predict conversion from MCI to AD in multiple prediction windows (up to 48 months) and averaged to predict progression to AD within 24-48 months	Demographics<br><br>Neuropsychiatric Assessments<br><br>Family Medical History<br><br>Comorbidities	AUROC:<br>0.857 (12 months);<br>0.858 (18 months);<br>0.980 (24 months);<br>0.964 (30 months);<br>0.922 (36 months);<br>0.948 (42 months);<br>0.975 (48 months).<br><br>Accuracy:<br>0.838 (12 months);<br>0.788 (18 months);<br>0.939 (24 months);<br>0.899 (30 months);<br>0.909 (36 months);<br>0.879 (42 months);<br>0.889 (48 months).
Orthogonal Latent space learning with Feature weighting and Graph learning Chen *et al.*	Distinguish between 1) normal cognition and MCI; 2) normal cognition vs. AD; 3) MCI that is stable vs. MCI that is progressive	Neuroimaging data	*No Longitudinal Prediction*<br><br>Classification Task 1:<br>AUROC: 0.719<br><br>Classification Task 2:<br>AUROC: 0.970<br><br>Classification Task 3:<br>AUROC: 0.814
Multi-kernel algorithm Hinrichs *et al.*	1) Classify AD patients vs. individuals with typical cognitive ability; 2) Predict which patients with MCI will progress to AD vs. reverting to typical cognition	Biological and cognitive markers	*No Longitudinal Prediction*<br><br>Classification Task 1:<br>Accuracy: 0.924<br><br>Classification Task 2:<br>AUROC: 0.9708
Logistic regression (LR) Marcisz and Polanska	Classify individuals as having MCI, AD, or normal cognition	Magnetic resonance imaging radiomic data	*No Longitudinal Prediction*<br><br>Highest AUROC:<br>Classification of AD<br>AUROC: 0.997<br><br>Lowest AUROC:<br>Classification of MCI vs. normal cognition<br>AUROC: 0.793

LR<br>Martínez-Torteya *et al*.	Classify 1) healthy controls vs. AD, 2) healthy controls vs. MCI, and MCI-AD	Imaging data<br><br>Clinical data<br><br>Biological data	*No Longitudinal Prediction*<br><br><u>Classification Task 1</u>:<br>AUROC: 0.945<br><br><u>Classification Task 2</u>:<br>AUROC: 0.864<br><br><u>Classification Task 3</u>:<br>AUROC: 0.838
Random forest<br>Massetti *et al.*	Progression from MCI to AD	Neuropsychological evaluation results<br><br>AD biomarkers	*No Longitudinal Prediction*<br><br><u>Accuracy</u>: 0.86
Random forest - highest accuracy<br>Mallo *et al.*	Risk of progression to dementia	Demographics<br><br>Comorbidities<br><br>Neuropsychiatric assessments	*No Longitudinal Prediction*<br><br><u>Accuracy</u>: 0.88
Network analysis<br>Morabito *et al.*	Progression of AD as measured by degeneration of connectivity density within the brains of patients with AD	Resting state electroencephalogram (EEG) data	*No Longitudinal Prediction*<br><br>Statistically significant ($p<0.05$) changes between initial assessment and follow-up at 3 months
Multivariate logistic regression<br>Peng et al.	Progression of MCI to AD	Positron-emission computed tomography (PET) scans<br><br>Multimodal data (systematic review)	*No Longitudinal Prediction*<br><br><u>AUROC</u>: 0.865
Multiple algorithmic approaches<br>Rutkowski *et al.*	Differentiate between patients with typical cognition vs. patients with MCI	EEG data derived from memory and visuospatial cognitive tasks	*No Longitudinal Prediction*<br><br><u>AUROC</u>: 0.49-1.00
Deep learning algorithm<br>Spasov *et al.*	Classify: 1) Patients with MCI that would progress to AD; 2) Patients with AD vs. typical cognition	Imaging<br><br>Demographics<br><br>Neuropsychological assessments<br><br>Genetic data	<u>Classification Task 1</u>:<br>AUROC: 0.925 for predicting MCI conversion to AD within 3 years<br><br><u>Classification Task 2</u>:<br>AUROC: 1.00

MCI = mild cognitive impairment; AUROC = area under the receiver operator characteristic curve

Chen Z, Liu Y, Zhang Y, Li Q; Alzheimer’s Disease Neuroimaging Initiative. Orthogonal latent space learning with feature weighting and graph learning for multimodal Alzheimer's disease diagnosis. Med Image Anal 2023 Feb;84:102698. https://doi.org/10.1016/j.media.2022.102698.
Hinrichs C, Singh V, Xu G, Johnson SC. Predictive markers for AD in a multi-modality framework: An analysis of MCI progression in the ADNI population. Neuroimage 2011;55:574–89. https://doi.org/10.1016/j.neuroimage.2010.10.081.

Marcisz A, Polanska J. Can T1-Weighted Magnetic Resonance Imaging Significantly Improve Mini-Mental State Examination-Based Distinguishing Between Mild Cognitive Impairment and Early-Stage Alzheimer's Disease? J Alzheimers Dis 2023;92(3):941-957. https://doi.org/10.3233/JAD-220806.

Martínez-Torteya A, Treviño V, Tamez-Peña JG. Improved Diagnostic Multimodal Biomarkers for Alzheimer's Disease and Mild Cognitive Impairment. Biomed Res Int 2015;2015:961314. https://doi.org/10.1155/2015/961314.

Massetti N, Russo M, Franciotti R, Nardini D, Mandolini GM, Granzotto A, et al. A Machine Learning-Based Holistic Approach to Predict the Clinical Course of Patients within the Alzheimer’s Disease Spectrum 1. J Alzheimers Dis 2022;85:1639–55. https://doi.org/10.3233/JAD-210573.

Mallo SC, Valladares-Rodriguez S, Facal D, Lojo-Seoane C, Fernández-Iglesias MJ, Pereiro AX. Neuropsychiatric symptoms as predictors of conversion from MCI to dementia:a machine learning approach. Int Psychogeriatr 2020;32:381–92. https://doi.org/10.1017/S1041610219001030.

Morabito FC, Campolo M, Labate D, Morabito G, Bonanno L, Bramanti A, et al. A Longitudinal EEG Study of Alzheimer’s Disease Progression Based on A Complex Network Approach. Int J Neural Syst 2015;25:1550005. https://doi.org/10.1142/S0129065715500057.

Peng J, Wang W, Qiaowei S, Jie H, Hui J, Xue Q, Zhongyu Y, Yuguo W, Zhenyu S, 18F-FDG-PET Radiomics Based on White Matter Predicts The Progression of Mild Cognitive Impairment to Alzheimer Disease: A Machine Learning Study. Acad Radiol 2023;30:1874-1884. https://doi.org/10.1016/j.acra.2022.12.033.

Rutkowski TM, Abe MS,Komendzinski T, Sugimoto H, Narebski S, Otake-Matsuura M. Machine learning approach for early onset dementia neurobiomarker using EEG network topology features. Front Hum Neurosci 2023;17. https://doi.org/10.3389/fnhum.2023.1155194

Spasov S, Passamonti L, Duggento A, Liò P, Toschi N. A parameter-efficient deep learning approach to predict conversion from mild cognitive impairment to Alzheimer’s disease. Neuroimage 2019;189:276–87. https://doi.org/10.1016/j.neuroimage.2019.01.03.

Table S2. Demographic information for training sets, for months 18, 30, 36, and 42 after baseline.

Demographics (Training Sets)		18 Months Non-AD (*n* = 313)	18 Months AD (*n*= 81)	30 Months Non-AD (*n* = 256)	30 Months AD (*n* = 138)	**36 Months** and **42 Months** (no change) Non-AD (*n* = 215)	**36 Months** and **42 Months** (no change) AD (*n* = 179)
Age (years)	55-60	9 (2.9%)	4 (4.9%)	8 (3.1%)	5 (3.6%)	8 (3.7%)	5 (2.8%)
	61-70	105 (33.5%)	13 (16.0%)	91 (35.5%)	27 (19.6%)	78 (36.3%)	40 (22.3%)
	71-80	147 (47.0%)	45 (55.6%)	114 (44.5%)	78 (56.5%)	100 (46.5%)	92 (51.4%)
	81-90	52 (16.6%)	19 (23.5%)	43 (16.8%)	28 (20.3%)	29 (13.5%)	42 (23.5%)
Sex Assigned at Birth	Female	119 (38.0%)	35 (43.2%)	99 (38.7%)	55 (39.9%)	78 (36.3%)	76 (42.5%)
	Male	194 (62.0%)	46 (56.8%)	157 (61.3%)	83 (60.1%)	137 (63.7%)	103 (57.5%)
Race	White	289 (92.3%)	79 (97.5%)	237 (92.6%)	131 (94.9%)	201 (93.5%)	167 (93.3%)
	Black or African American	11 (3.5%)	1 (1.2%)	9 (3.5%)	3 (2.2%)	5 (2.3%)	7 (3.9%)
	Asian	8 (2.6%)	1 (1.2%)	6 (2.3%)	3 (2.2%)	5 (2.3%)	4 (2.2%)
	American Indian or Alaskan Native	1 (0.3%)	0 (0.0%)	1 (0.3%)	0 (0.0%)	1 (0.4%)	0 (0.0%)
	More than one race	4 (1.3%)	0 (0.0%)	3 (1.2%)	1 (0.7%)	3 (1.4%)	1 (0.6%)
Ethnicity	Hispanic/Latino	11 (3.5%)	1 (1.2%)	9 (3.5%)	3 (2.2%)	6 (2.8%)	6 (3.4%)
	Not Hispanic/Latino	302 (96.5%)	80 (98.8%)	247 (96.5%)	135 (97.8%)	209 (97.2%)	173 (96.6%)
Comorbidities	Diabetes	28 (8.9%)	6 (7.4%)	24 (9.4%)	10 (7.2%)	21 (9.8%)	13 (7.3%)
	Depression	99 (31.6%)	24 (29.6%)	83 (32.4%)	40 (29.0%)	69 (32.1%)	54 (30.2%)
	Osteoporosis or Osteoarthritis	75 (24.0%)	17 (21.0%)	62 (24.2%)	30 (21.7%)	52 (24.2%)	40 (22.3%)
	Cerebrovascular Disease	14 (4.5%)	3 (3.7%)	13 (5.1%)	4 (2.9%)	9 (4.2%)	8 (4.5%)
	Hypertension	131 (41.9%)	37 (45.7%)	107 (41.8%)	61 (44.2%)	88 (40.9%)	80 (44.6%)

	Hearing or vision impairment	80 (25.6%)	15 (18.5%)	63 (24.6%)	32 (23.2%)	56 (26.0%)	39 (21.8%)
	Coronary heart disease	16 (5.1%)	1 (1.2%)	14 (5.5%)	3 (2.2%)	11 (5.1%)	6 (3.4%)

Supplemental Table S3. Demographic information for hold-out test sets, for months 12, 24, and 48 after baseline.

Demographics (Test Sets)		12 Months		24 Months		48 Months	
		**Non-AD (*n*= 89)**	**AD (*n* = 10)**	**Non-AD (*n* = 60)**	**AD (*n* = 39)**	**Non-AD (*n* = 44)**	**AD (*n* = 55)**
Age (years)	55-60	8 (9.0%)	0 (0%)	4 (6.7%)	4 (10.3%)	4 (9.1%)	4 (7.3%)
	61-70	29 (32.6%)	5 (50.0%)	20 (33.3%)	14 (35.9%)	16 (36.4%)	18 (32.7%)
	71-80	41 (46.1%)	3 (30.0%)	29 (48.3%)	15 (38.5%)	18 (40.9%)	26 (47.3%)
	81-90	11 (12.4%)	2 (20.0%)	7 (11.7%)	6 (10.3%)	6 (13.6%)	7 (12.7%)
Sex Assigned at Birth	Female	35 (39.3%)	5 (50.0%)	20 (33.3%)	20 (51.3%)	15 (34.1%)	25 (45.5%)
	Male	54 (60.7%)	5 (50.0%)	40 (66.7%)	19 (48.7%)	29 (65.9%)	30 (54.5%)
Race	White	89 (100.0%)	10 (100.0%)	60 (100.0%)	39 (100.0%)	44 (100.0%)	55 (100.0%)
	Black or African American	0 (0.0%)	0 (0.0%)	0 (0.0%)	0 (0.0%)	0 (0.0%)	0 (0.0%)
	Asian	0 (0.0%)	0 (0.0%)	0 (0.0%)	0 (0.0%)	0 (0.0%)	0 (0.0%)
	American Indian or Alaskan Native	0 (0.0%)	0 (0.0%)	0 (0.0%)	0 (0.0%)	0 (0.0%)	0 (0.0%)
	More than one race	0 (0.0%)	0 (0.0%)	0 (0.0%)	0 (0.0%)	0 (0.0%)	0 (0.0%)
Ethnicity	Hispanic/ Latino	3 (3.4%)	0 (0.0%)	1 (1.7%)	2 (5.1%)	1 (2.3%)	2 (3.6%)
	Not Hispanic/ Latino	86 (96.7%)	10 (100.0%)	59 (98.3%)	37 (94.9%)	43 (97.7%)	53 (96.4%)
Comorbidities	Diabetes	9 (10.1%)	2 (20.0%)	6 (10.0%)	5 (12.8%)	4 (9.1%)	7 (12.7%)
	Depression	30 (33.7%)	6 (60.0%)	18 (30.0%)	18 (46.2%)	13 (29.5%)	23 (41.8%)
	Osteoporosis or Osteo-arthritis	21 (23.6%)	2 (20.0%)	13 (21.7%)	10 (25.6%)	8 (18.2%)	15 (27.3%)

	Cerebro-vascular Disease	2 (2.2%)	1 (10.0%)	1 (1.7%)	2 (5.1%)	0 (0%)	3 (5.5%)
	Hypertension	32 (36.0%)	2 (20.0%)	23 (38.3%)	11 (28.2%)	18 (40.9%)	16 (29.1%)
	Hearing or vision impairment	14 (15.7%)	0 (0.0%)	7 (11.7%)	7 (17.9%)	4 (9.1%)	10 (18.2%)
	Coronary heart disease	2 (2.2%)	0 (0.0%)	1 (1.7%)	1 (2.6%)	1 (2.3%)	1 (1.8%)

Supplemental Table S4. Demographic information for hold-out test sets, for months 18, 30, 36, and 42 after baseline.

**Demographics (Test Sets)**		**18 Months**		**30 Months**		**36 Months**		**42 Months**	
		**Non-AD (*n*= 79)**	**AD (*n* = 20)**	**Non-AD (*n* = 59)**	**AD (*n* = 40)**	**Non-AD (*n* = 49)**	**AD (*n* = 50)**	**Non-AD (*n* = 48)**	**AD (*n* = 51)**
Age (years)	55-60	7 (8.9%)	1 (5.0%)	4 (6.8%)	4 (10.0%)	4 (8.2%)	4 (8.0%)	4 (8.3%)	4 (7.8%)
	61-70	27 (34.2%)	7 (35.0%)	19 (32.2%)	15 (37.5%)	19 (38.8%)	15 (30.0%)	18 (37.5%)	16 (31.4%)
	71-80	38 (48.1%)	6 (30.0%)	29 (49.2%)	15 (37.5%)	19 (38.8%)	25 (50.0%)	19 (39.6%)	25 (49.0%)
	81-90	7 (8.9%)	6 (30.0%)	7 (11.9%)	6 (10.0%)	7 (14.3%)	6 (12.0%)	7 (14.6%)	6 (11.8%)
Sex Assigned at Birth	Female	30 (38.0%)	10 (50.0%)	20 (33.9%)	20 (50.0%)	17 (34.7%)	23 (46.0%)	17 (35.4%)	23 (45.1%)
	Male	49 (62.0%)	10 (50.0%)	39 (66.1%)	20 (50.0%)	32 (65.3%)	27 (54.0%)	31 (64.6%)	28 (54.9%)
Race	White	79 (100.0%)	20 (100.0%)	58 (100.0%)	40 (100.0%)	49 (100.0%)	50 (100.0%)	48 (100.0%)	51 (100.0%)
	Black or African American	0 (0.0%)	0 (0.0%)	0 (0.0%)	0 (0.0%)	0 (0.0%)	0 (0.0%)	0 (0.0%)	0 (0.0%)
	Asian	0 (0.0%)	0 (0.0%)	0 (0.0%)	0 (0.0%)	0 (0.0%)	0 (0.0%)	0 (0.0%)	0 (0.0%)
	American Indian or Alaskan Native	0 (0.0%)	0 (0.0%)	0 (0.0%)	0 (0.0%)	0 (0.0%)	0 (0.0%)	0 (0.0%)	0 (0.0%)
	More than one race	0 (0.0%)	0 (0.0%)	0 (0.0%)	0 (0.0%)	0 (0.0%)	0 (0.0%)	0 (0.0%)	0 (0.0%)
Ethnicity	Hispanic/ Latino	2 (2.5%)	1 (5.0%)	1 (1.7%)	2 (5.0%)	1 (2.0%)	2 (4.0%)	1 (2.1%)	2 (3.9%)
	Not Hispanic/ Latino	77 (97.5%)	19 (95.0%)	58 (98.3%)	38 (95.0%)	48 (98.0%)	48 (96.0%)	47 (97.9%)	49 (96.1%)
Comor-bidities	Diabetes	9 (11.4%)	2 (10.0%)	6 (10.2%)	5 (12.5%)	4 (8.2%)	7 (14.0%)	4 (8.3%)	7 (13.7%)
	Depression	26 (32.9%)	10 (50.0%)	18 (30.5%)	18 (45.0%)	15 (30.6%)	21 (42.0%)	15 (31.3%)	21 (41.2%)
	Osteoporosis or Osteoarthritis	17 (21.5%)	6 (30.0%)	13 (22.0%)	10 (25.0%)	10 (20.4%)	13 (26.0%)	10 (20.8%)	13 (25.5%)

	Cerebro-vascular Disease	2 (2.5%)	1 (5.0%)	0 (0.0%)	3 (7.5%)	0 (0.0%)	3 (6.0%)	0 (0.0%)	3 (5.9%)
	Hypertension	31 (39.2%)	3 (15.0%)	22 (37.3%)	12 (30.0%)	18 (36.7%)	16 (32.0%)	18 (37.5%)	16 (31.4%)
	Hearing or vision impairment	13 (16.5%)	1 (5.0%)	7 (11.9%)	7 (17.5%)	4 (8.2%)	10 (20.0%)	4 (8.3%)	10 (19.6%)
	Coronary heart disease	1 (1.3%)	1 (5.0%)	1 (1.7%)	1 (2.5%)	1 (2.0%)	1 (2.0%)	1 (2.1%)	1 (2.0%)

Supplemental Figure S1. The receiver operating characteristic (ROC) curves for the Alzheimer's disease (AD) class vs. the non-AD class as given by the Mini-Mental State Examination (MMSE) classifier when the prediction algorithm is tested on the hold-out test sets, for each of the seven prediction windows showing performance of the machine learning algorithm (MLA) and the MMSE over time.

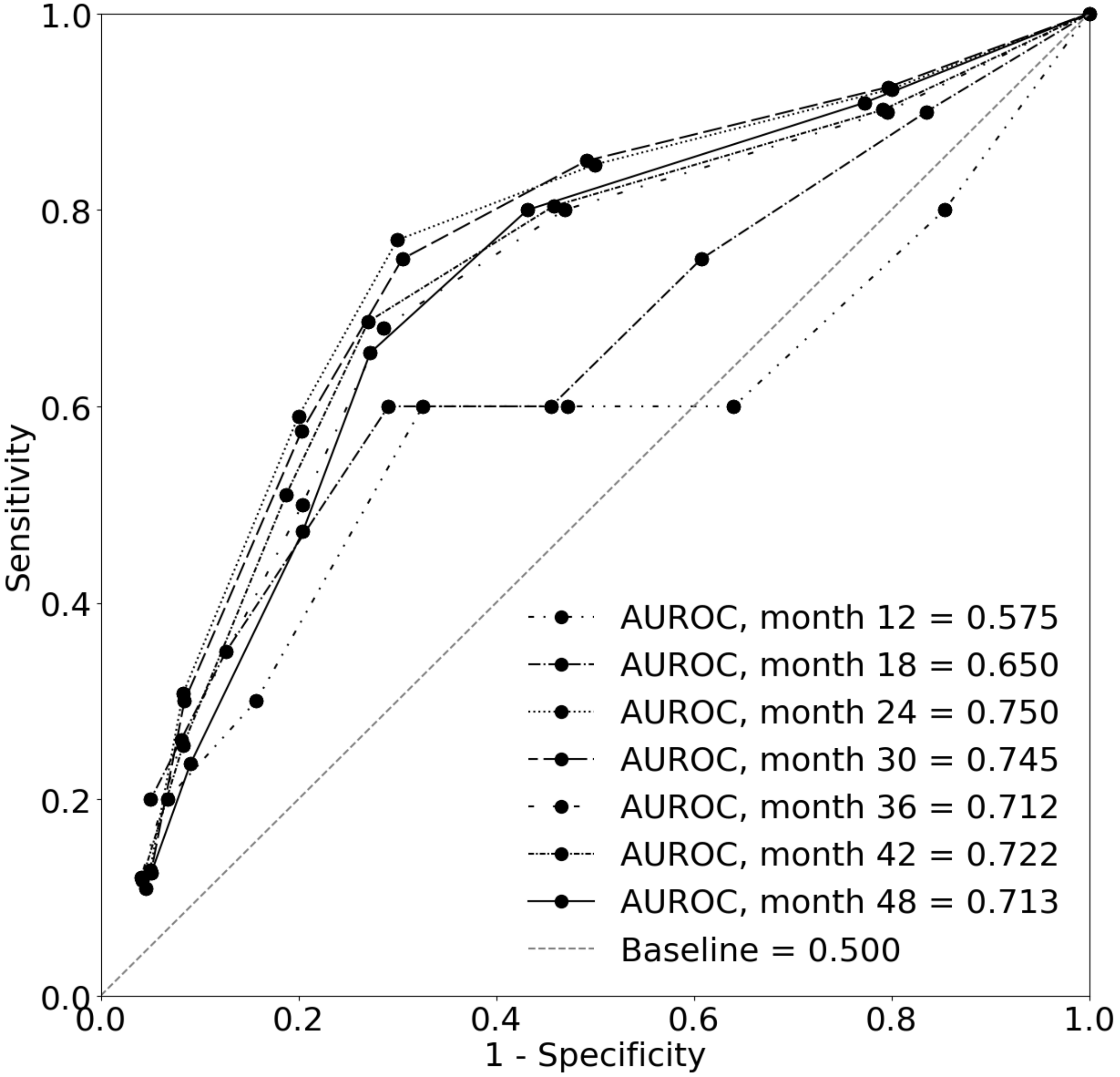

Supplemental Figure S2. The receiver operating characteristic (ROC) curves for the Alzheimer's disease (AD) class vs. the non-AD class as given by the machine learning algorithm (MLA) and the Mini-Mental State Examination (MMSE) classifier showing performance of the MLA and the MMSE over time: (A) 18-month prediction window; (B) 30-month prediction window; (C) 36-month prediction window; (D) 42-month prediction window.

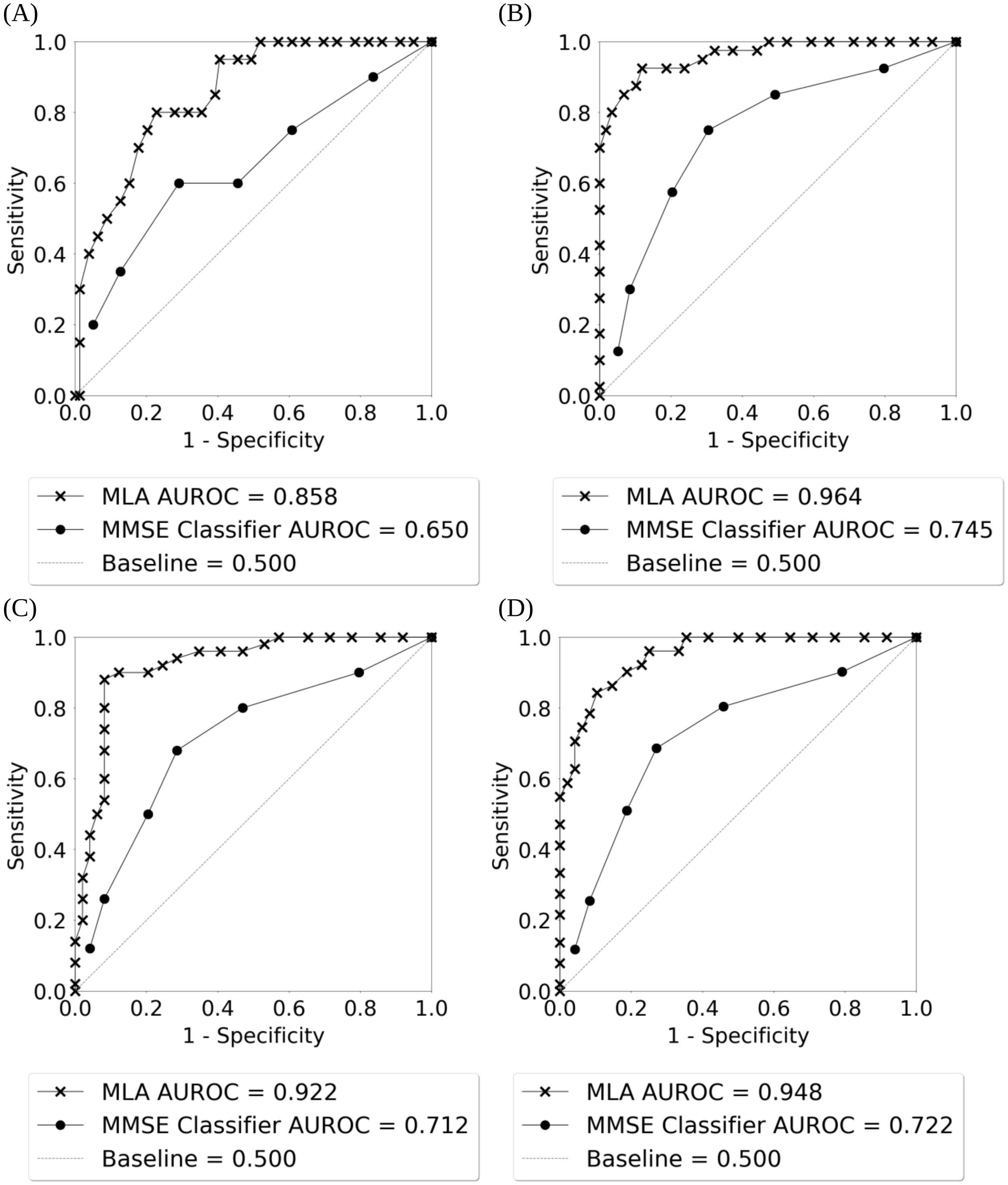

Supplemental Figure S3. The receiver operating characteristic (ROC) curves for the Alzheimer's disease (AD) class vs. the non-AD class as given by the machine learning algorithm (MLA) classifier when the prediction algorithm is tested on the hold-out test sets for each of the seven prediction windows showing performance of the MLA over time.

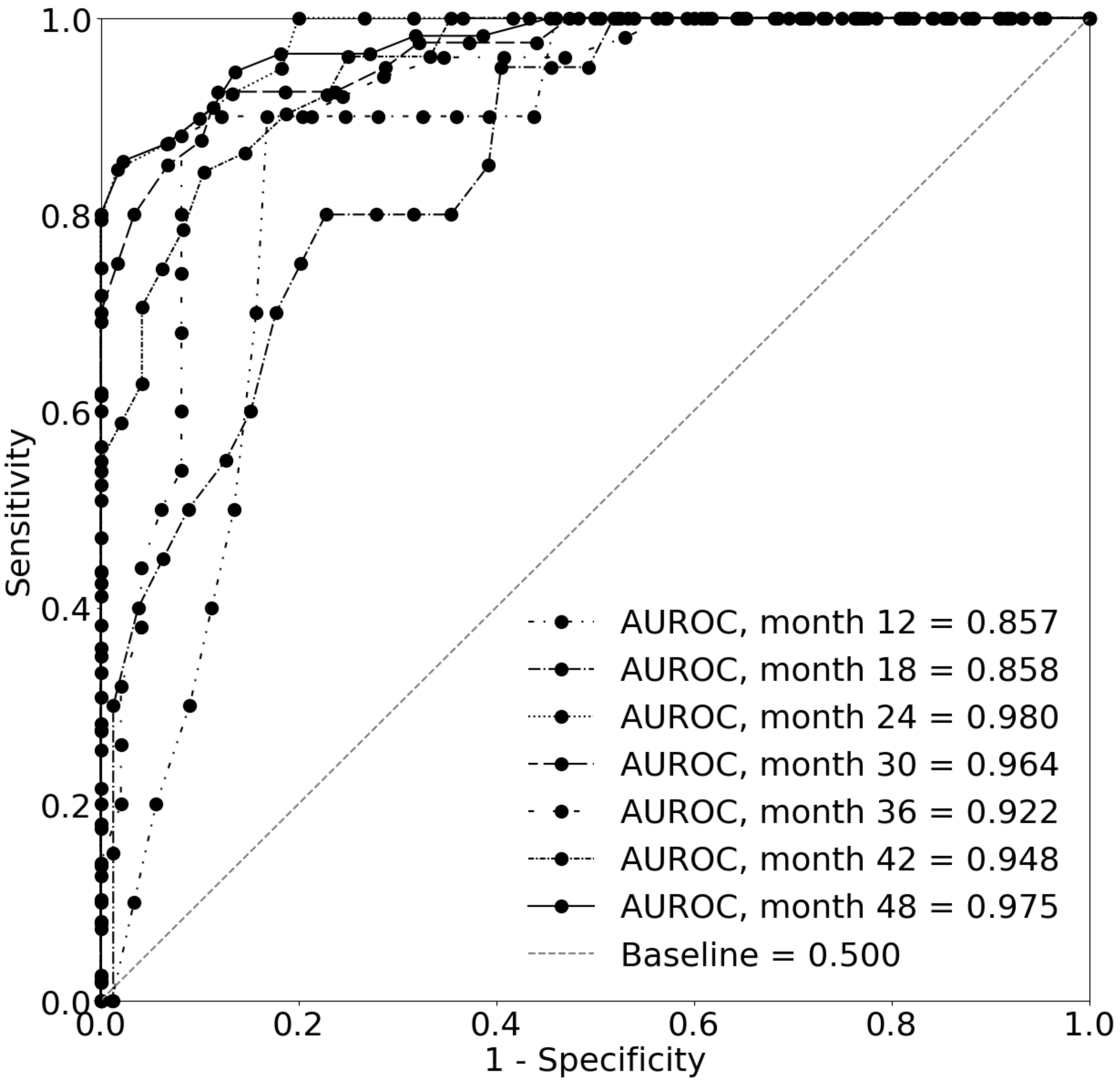

Supplemental Figure S4. The positive predictive value (PPV) (A), negative predictive value (NPV) (B), and accuracy (C) vs. prediction window for the machine learning algorithm (MLA) and Mini-Mental State Examination (MMSE) classifiers across all seven prediction windows showing performance of the MLA and the MMSE over time.

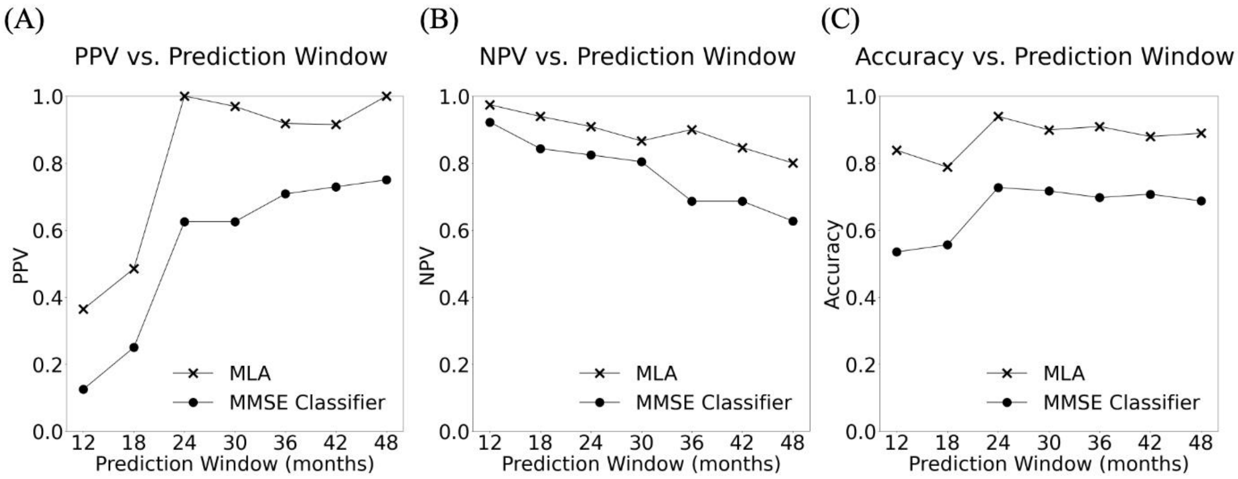

Supplemental Table S5. Detailed feature inputs for all input categories.

Neuropsychological Assessments	
**<u>Alzheimer's Disease Assessment Scale (ADAS)</u>**<br>• Word Recall<br>• Commands<br>• Construction<br>• Delayed Word Recall<br>• Naming<br>• Ideational Praxis<br>• Orientation<br>• Word Recognition<br>• Recall Instructions<br>• Spoken Language<br>• Word Finding Difficulty<br>• Comprehension<br>• Number Cancellation<br><br>**<u>Mini-Mental State Examination (MMSE)</u>**<br>• What is today's date?<br>• What is the year?<br>• What is the month?<br>• What day of the week is today?<br>• What season is it?<br>• What is the name of this place?<br>• What floor are we on?<br>• What town or city are we in?<br>• What county are we in?<br>• What state are we in?<br>• Immediately repeat "ball"<br>• Immediately repeat "flag"<br>• Immediately repeat "tree"<br>• Number of trials for accurate immediate repetition of ball/flag/tree<br>• "D" when spelling "WORLD" backwards<br>• "L" when spelling "WORLD" backwards<br>• "R" when spelling "WORLD" backwards<br>• "O" when spelling "WORLD" backwards<br>• "W" when spelling "WORLD" backwards<br>• Delayed recall of "ball"	**<u>Functional Activities Questionnaire (FAQ)</u>**<br>• Finance score<br>• Forms score<br>• Shopping score<br>• Game score<br>• Beverage score<br>• Meal score<br>• Events score<br>• Media question<br>• Remembering question<br>• Travel question<br>• Total Score<br><br>**<u>Neuropsychiatric Inventory Questionnaire (NPI-Q)</u>**<br>• Delusion question<br>• Severity of delusions<br>• Hallucination question<br>• Severity of hallucinations<br>• Agitation/Aggression question<br>• Severity of Agitation/Aggression<br>• Depression/Dysphoria question<br>• Severity of Depression/Dysphoria<br>• Anxiety question<br>• Severity of Anxiety<br>• Elation/Euphoria question<br>• Severity of Elation/Euphoria<br>• Apathy/Indifference question<br>• Severity of Apathy/Indifference<br>• Disinhibition question<br>• Severity of Disinhibition<br>• Irritability/Lability question<br>• Severity of Irritability/Lability<br>• Aberrant Motor Behavior question<br>• Severity of Aberrant Motor Behavior<br>• Sleep question<br>• Severity of Sleep<br>• Appetite question<br>• Severity of Appetite<br>• Total Score

• Delayed recall of "flag"<br>• Delayed recall of "tree"<br>• Identification of a watch<br>• Identification of a pencil<br>• Repetition of "no ifs, ands, or buts"<br>• Take paper in right hand<br>• Fold paper in half<br>• Put paper on floor<br>• Read "Close your eyes" and close eyes<br>• Write a sentence<br>• Copying of a design<br>• Total Score<br><br>**Clinical Dementia Rating (CDR)**<br>• Memory score<br>• Orientation score<br>• Judgment & Problem Solving score<br>• Community Affairs score<br>• Home & Hobbies score<br>• Personal Care score<br>• Global score<br>• Sum-of-Boxes score<br><br>**Neuropsychological Assessment Battery (NAB)**<br>• Delayed Recall Total<br>• WAIS-R Digit Symbol<br>• Trails B Score<br><br>**Modified Hachinski Ischemic Score**<br>• Abrupt onset of dementia<br>• Stepwise deterioration of dementia<br>• Somatic complaints<br>• Emotional incontinence<br>• History of hypertension<br>• History of stroke<br>• Focal neurologic symptoms<br>• Focal neurologic signs<br>• Total Score	**Rey Auditory Verbal Learning Test (RAVLT)**<br>• Forgetting score (trial 5)<br>• Immediate score (sum of 5 trials)<br>• Learning score (trial 5 – trial 1)<br>• Percent forgetting score<br><br>**ADNI-specific composite scoring**<br>• Memory composite score<br>• Executive functioning composite score<br><br>**Geriatric Depression Scale (GDS)**<br>• Are you basically satisfied with your life?<br>• Have you dropped many of your interests?<br>• Emptiness question<br>• Bored question<br>• Spirits question<br>• Fear question<br>• Happiness question<br>• Helplessness question<br>• Home question<br>• Memory question<br>• Living question<br>• Worthlessness question<br>• Energy question<br>• Hopelessness question<br>• Better question<br>• Total Score
**Comorbidities**	**Demographics**
• Any type of diabetes mellitus<br>• Type 2 diabetes mellitus<br>• Hypertension<br>• Stroke<br>• Hearing impairment	**Age** (years)<br>**Education** (years)<br>**Sex Assigned at Birth**<br>• Female

<table>
<tr><td>

- Vision impairment
- Osteoporosis
- Cardiovascular Disease

</td><td rowspan="3">

- Male

<u>Ethnicity</u>

- Hispanic
- Non-Hispanic
- Ethnicity Unknown

<u>Race</u>

- African American / Black
- Asian
- Mixed Race
- Native American
- Pacific Islander
- White / Caucasian
- Race Unknown

<u>Marital Status</u>

- Divorced
- Married
- Never married
- Marital status unknown
- Widowed

</td></tr>
<tr><td>Family Medical History</td></tr>
<tr><td>

- Mother's dementia status
- Mother's Alzheimer's disease status
- Father's dementia status
- Father's Alzheimer's disease status
- Siblings' dementia status
- Siblings' Alzheimer's disease status

</td></tr>
</table>

Supplemental Table S6. Machine learning algorithm (MLA) performance on corresponding hold-out test sets for the 18-, 30-, 36-, and 42-month prediction windows showing performance of the XGBoost-based MLA, the multi-layer perceptron (MLP) neural network, *k*-nearest neighbors (KNN), and logistic regression (LR) models, and the Mini-Mental State Examination (MMSE) over time. XGBoost is a widely used gradient-boosted tree ensemble method.

		**Performance Metrics**					
**Prediction Window**	**Modeling Approach**	**AUROC (95% CI)**	**Sensitivity (95% CI)**	**Specificity (95% CI)**	**PPV (95% CI)**	**NPV (95% CI)**	**Accuracy (95% CI)**
18 months	MLA	0.858 (0.778-0.931)	0.800 (0.703-0.897)	0.785 (0.735-0.835)	0.485 (0.390-0.579)	0.939 (0.907-0.971)	0.788 (0.743-0.833)
	LR	0.812 (0.680 - 0.915)	0.800 (0.733 - 0.867)	0.733 (0.690 - 0.776)	0.500 (0.434 - 0.566)	0.917 (0.887 - 0.947)	0.750 (0.714 - 0.786)
	MLP	0.842 (0.741-0.928)	0.800 (0.685-0.915)	0.733 (0.701-0.765)	0.500 (0.431-0.569)	0.917 (0.882-0.952)	0.538 (0.419-0.656)
	KNN	0.838 (0.739-0.924)	0.950 (0.931-0.969)	0.550 (0.525-0.575)	0.413 (0.385-0.441)	0.971 (0.959-0.982)	0.650 (0.629-0.671)
	MMSE Classifier ‡	0.650 (0.496-0.803)	0.600 (0.367-0.833)	0.544 (0.425-0.664)	0.250 (0.117-0.383)	0.843 (0.735-0.952)	0.556 (0.449 0.662)
30 months	MLA	0.964 (0.924-0.991)	0.775 (0.675-0.875)	0.983 (0.958-1.000)	0.969 (0.922-1.000)	0.866 (0.803-0.929)	0.899 (0.853-0.945)
	LR	0.966 (0.927-0.993)	0.795 (0.708-0.881)	0.977 (0.947-1.000)	0.969 (0.928-1.000)	0.843 (0.775-0.911)	0.892 (0.846-0.937)
	MLP	0.958 (0.913-0.993)	0.872 (0.787-0.956)	0.977 (0.942-1.000)	0.971 (0.927-1.000)	0.896 (0.826-0.965)	0.928 (0.883-0.973)
	KNN	0.925 (0.853-0.983)	0.846 (0.777-0.915)	0.932 (0.887 - 0.977)	0.917 (0.862-0.972)	0.872 (0.814-0.930)	0.892 (0.851-0.932)
	MMSE Classifier ‡	0.745 (0.638-0.843)	0.750 (0.604-0.896)	0.695 (0.567-0.822)	0.625 (0.476-0.774)	0.804 (0.686-0.922)	0.717 (0.621-0.813)

36 months	MLA	0.922 (0.863-0.972)	0.900 (0.857-0.943)	0.918 (0.878-0.958)	0.918 (0.848-0.958)	0.900 (0.857-0.943)	0.909 (0.879-0.939)
	LR	0.913 (0.843-0.973)	0.842 (0.766-0.918)	0.929 (0.878-0.979)	0.914 (0.854-0.975)	0.867 (0.802-0.931)	0.888 (0.842-0.933)
	MLP	0.932 (0.865-0.982)	0.842 (0.823-0.861)	0.905 (0.890-0.919)	0.889 (0.872-0.905)	0.864 (0.847-0.880)	0.875 (0.863-0.887)
	KNN	0.880 (0.796-0.952)	0.816 (0.741-0.891)	0.857 (0.793-0.921)	0.838 (0.766-0.910)	0.837 (0.770-0.904)	0.838 (0.788-0.887)
	MMSE Classifier ‡	0.712 (0.602-0.811)	0.680 (0.540-0.820)	0.714 (0.577-0.852)	0.708 (0.569-0.848)	0.686 (0.548-0.825)	0.697 (0.599-0.795)
42 months	MLA	0.948 (0.905-0.981)	0.843 (0.777-0.909)	0.917 (0.865-0.969)	0.915 (0.862-0.968)	0.846 (0.781-0.911)	0.879 (0.836-0.921)
	LR	0.941 (0.894-0.978)	0.784 (0.711-0.858)	0.938 (0.893-0.982)	0.930 (0.881-0.980)	0.804 (0.736-0.871)	0.859 (0.814-0.903)
	MLP	0.852 (0.776-0.918)	0.784 (0.779-0.790)	0.792 (0.786-0.797)	0.800 (0.794-0.806)	0.776 (0.770-0.781)	0.788 (0.784-0.792)
	KNN	0.885 (0.811-0.948)	0.804 (0.738-0.870)	0.771 (0.699-0.843)	0.788 (0.721-0856)	0.787 (0.716-0.858)	0.788 (0.739-0.837)
	MMSE Classifier ‡	0.722 (0.615-0.822)	0.686 (0.548-0.825)	0.729 (0.593-0.866)	0.729 (0.593-0.866)	0.686 (0.548-0.825)	0.707 (0.610-0.804)

‡ Note: The MMSE classifier uses a threshold score value of 27. AUROC = area under the receiver operator characteristic curve; CI = confidence interval; PPV = positive predictive value; NPV = negative predictive value

Supplemental Figure S5. Bar plot visualization of averaged machine learning algorithm (MLA) performance in comparison with the performance of the Mini-Mental State Examination (MMSE) classifier at 48 months. Each pair of bars represents a different performance metric: area under the receiver operating characteristic curve (AUROC), sensitivity, specificity, positive predictive value (PPV), negative predictive value (NPV), and accuracy. The value on each bar represents the calculated metric for either the MLA or MMSE classifier, and the error bars at the top of each bar represent 95% confidence intervals.

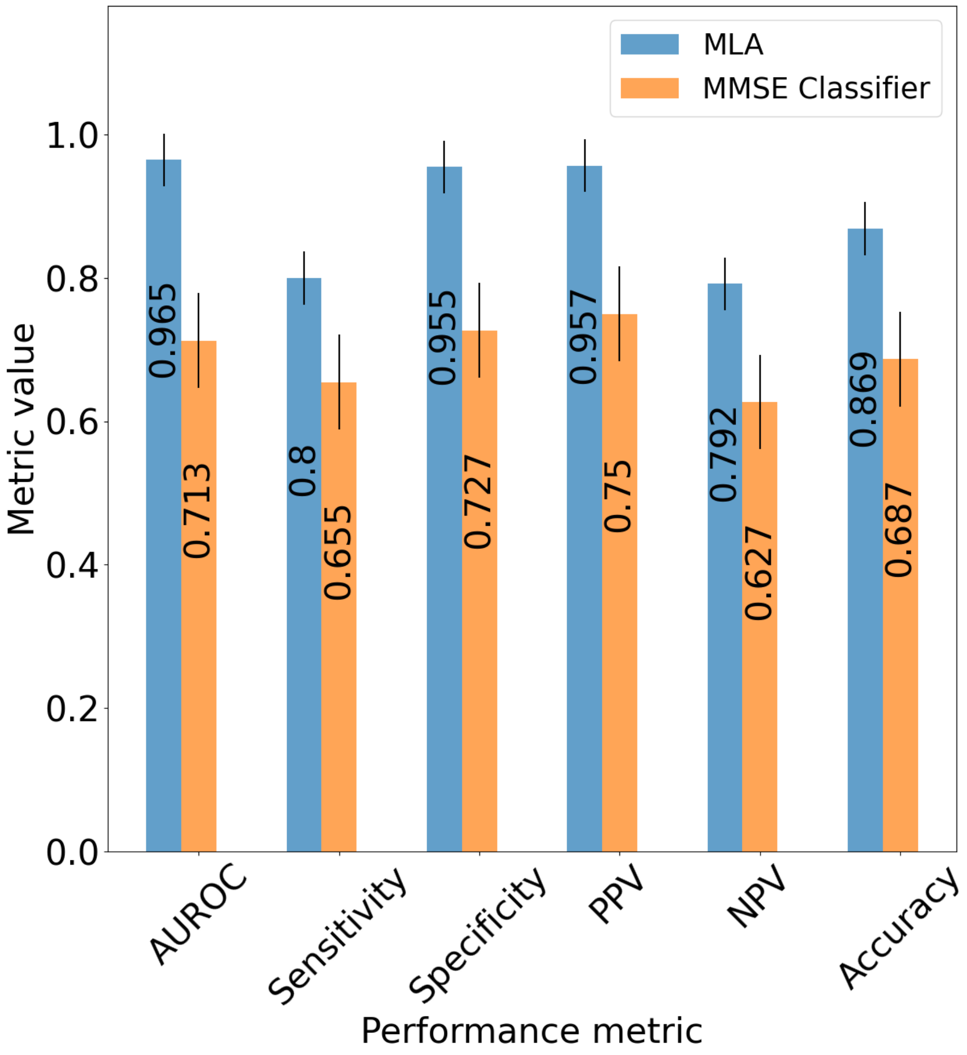

Supplemental Table S7. The top 10 features impacting the performance of the machine learning algorithm (MLA) at the 18-, 30-, 36-, and 42-month prediction windows.

**18 months**	**30 months**	**36 months**	**42 months**
1. FAQ total score	1. ADAS-Cog-11 total score	1. ADAS-Cog-11 Word Recall	1. RAVLT Forgetting subscore
2. Ethnicity	2. Ethnicity	2. FAQ total score	2. RAVLT Immediate Recall subscore
3. MMSE flag	3. FAQ total score	3. ADAS-Cog-11 total score	3. RAVLT Percent Forgetting
4. FAQ Tax Forms subscore	4. ADAS-Cog-11 Word Recall	4. FAQ Tax Forms subscore	4. ADAS-Cog-13 Delayed Word Recall
5. MMSE tree	5. ADAS-Cog-11 Orientation	5. MMSE total score	5. FAQ Remembering subscore
6. FAQ Remembering subscore	6. FAQ Attention and Understanding subscore	6. RAVLT total score	6. ADAS-Cog-11 total score
7. ADAS-Cog-11 total score	7. FAQ Current Events subscore	7. MMSE tree	7. FAQ total score
8. MMSE letter “W” in “spell WORLD backwards” task	8. RAVLT Immediate Recall subscore	8. RAVLT Immediate Recall subscore	8. ADAS-Cog-11 Word Recognition
9. Logical Memory Delayed Recall total score	9. NPIQ time to complete	9. RAVLT Forgetting subscore	9. FAQ Tax Forms subscore
10. NPIQ total score	10. CDR Community Affairs score	10. RAVLT Percent Forgetting	10. RAVLT Learning subscore

FAQ = Functional Assessment Questionnaire; MMSE = Mini-Mental State Examination; ADAS-Cog-11 = Alzheimer’s Disease Assessment Scale–Cognitive Subscale (11 tasks); NPIQ = Neuropsychiatric Inventory–Questionnaire; CDR = Clinical Dementia Rating; RAVLT = Rey Auditory Verbal Learning Test; ADAS-Cog-13 = Alzheimer’s Disease Assessment Scale–Cognitive Subscale (13 tasks)
